# Comparative transcriptome and metabolome analysis revealed diversity in the response of resistant and susceptible rose (*Rosa hybrida*) varieties to *Marssonina rosae*

**DOI:** 10.3389/fpls.2024.1362287

**Published:** 2024-02-22

**Authors:** Jurong Song, Feng Chen, Bo Lv, Cong Guo, Jie Yang, Jiaqi Guo, Li Huang, Guogui Ning, Yuanyuan Yang, Fayun Xiang

**Affiliations:** ^1^ Cash Crops Research Institute, Hubei Academy of Agricultural Sciences, Wuhan, China; ^2^ National Key Laboratory for Germplasm Innovation & Utilization of Horticultural Crops, Huazhong Agricultural University, Wuhan, China

**Keywords:** rose, black spot disease, transcriptome, metabolome, defense response, brassinosteroid

## Abstract

Rose black spot disease caused by *Marssonina rosae* is among the most destructive diseases that affects the outdoor cultivation and production of roses; however, the molecular mechanisms underlying the defensive response of roses to *M. rosae* have not been clarified. To investigate the diversity of response to *M. rosae* in resistant and susceptible rose varieties, we performed transcriptome and metabolome analyses of resistant (KT) and susceptible (FG) rose varieties and identified differentially expressed genes (DEGs) and differentially accumulated metabolites (DAMs) in response to *M. rosae* at different time points. In response to *M. rosae*, DEGs and DAMs were mainly upregulated compared to the control and transcription factors were concentrated in the WRKY and AP2/ERF families. Gene Ontology analysis showed that the DEGs of FG were mainly enriched in biological processes, such as the abscisic acid-activated signaling pathway, cell wall, and defense response, whereas the DEGs of KT were mainly enriched in Golgi-mediated vesicle transport processes. Kyoto Encyclopedia of Genes and Genomes analysis showed that the DEGs of both varieties were concentrated in plant–pathogen interactions, plant hormone signal transduction, and mitogen-activated protein kinase signaling pathways, with the greatest number of DEGs associated with brassinosteroid (BR) in the plant hormone signal transduction pathway. The reliability of the transcriptome results was verified by qRT-PCR. DAMs of KT were significantly enriched in the butanoate metabolism pathway, whereas DAMs of FG were significantly enriched in BR biosynthesis, glucosinolate biosynthesis, and tryptophan metabolism. Moreover, the DAMs in these pathways were significantly positively correlated with the DEGs. Disease symptoms were aggravated when FG leaves were inoculated with *M. rosae* after 24-epibrassinolide treatment, indicating that the response of FG to *M. rosae* involves the BR signaling pathway. Our results provide new insights into the molecular mechanisms underlying rose response to *M. rosae* and lay a theoretical foundation for formulating rose black spot prevention and control strategies and cultivating resistant varieties.

## Introduction

As important ornamental plants, roses (*Rosa* spp.) are widely sold as cut flowers, garden plants, and potted plants and have important economic value and cultural connotations (Hibrand Saint-Oyant et al., 2018). However, when this plant is produced and cultivated outdoors, the parts dominated by leaves may be damaged by black spot disease. The disease produces black spots with irregular edges. In the later stages, yellowing occurs around the edges of the black spots area, resulting in the leaves falling off prematurely. Such changes greatly reduce the ornamental value of roses, decrease the vitality of susceptible plants, and may even lead to death (Gachomo and Kotchoni, 2007; Debener, 2019). *Marssonina rosae* is the pathogen of rose black spot disease. This fungus is a hemibiotrophic ascomycete that exclusively parasitizes *Rosa* plants, and its sexual stage is *Diplocarpon rosae* (Gachomo et al., 2006; Whitaker et al., 2007; Debener, 2019). As a worldwide disease, rose black spot was one of the factors underlying a 10% drop in the sales of French garden roses in 2017 (Lopez Arias et al., 2020). It was also one of the major diseases that reduced the production value of American gardens from $203 million in 2014 to $168 million in 2019 (Rawandoozi et al., 2022). Rose black spot is mainly controlled by chemical agents, which not only increase economic and labor costs but also contaminate the environment and cause drug resistance in pathogens (Byrne et al., 2019; Zurn et al., 2020). Moreover, this practice is contrary to the green and sustainable development of agricultural production. Planting resistant varieties may be the most economical, effective, and environmentally friendly way to control black spot in roses. The results of market surveys have also shown that the demand for resistant varieties is increasing among rose growers and consumers (Waliczek et al., 2015; Byrne et al., 2019). However, the effective breeding of resistant varieties depends on molecular studies of resistance.

Unlike humans, plants do not have specialized immune cells or organs or adaptive immune systems. However, during their long-term coevolution with pathogens, plants have evolved an innate immune system to recognize and defend against pathogen infections (Ausubel, 2005; Jones and Dangl, 2006). Membrane-localized pattern recognition receptors (PRRs) and intracellular nucleotide-binding and leucine-rich repeat receptors (NLRs) mediate the two-layer innate immune system in plants (Jones and Dangl, 2006). PPRs typically include receptor-like kinases or receptor-like proteins that trigger immunity by recognizing pathogen-associated molecular patterns (PAMPs), i.e., PAMP-triggered immunity (PTI) (Jones and Dangl, 2006; Zipfel, 2014). PAMPs contain bacterial flagellin, elongation factor Tu, and fungal chitin (Zipfel, 2014), which are hightly conserved in different types of pathogens. Therefore, plant PTI belongs to a broad spectrum of defense mechanisms. However, pathogens secrete effectors that interfere with or inhibit PTI, thereby increasing plant susceptibility. Plants recognize pathogenic effectors directly or indirectly through NLRs, thereby triggering a second layer of immunity, i.e., effector-triggered immunity (ETI) (Jones and Dangl, 2006; Lolle et al., 2020). During evolution, pathogens may have escaped the ETI of plants and caused plant susceptibility. In general, ETI induces a more intense immune response than PTI. A complex interaction likely occurs between ETI and PTI, and both can cause overlapping downstream immune responses, such as the mitogen-activated protein kinase (MAPK) cascade, Ca^2+^, reactive oxygen species burst, and plant hormone signal transduction (Lolle et al., 2020; Yuan et al., 2021).

Studies on the molecular mechanisms underlying resistance to *M. rosae* infection in roses have focused on the mapping of resistance genes, including qualitative trait loci for vertical resistance and quantitative trait loci (QTLs) for horizontal resistance. Varying progress has been made in mapping these four single gene disease resistance loci, namely *Rdr1* (von Malek and Debener, 1998; Biber et al., 2009; Menz et al., 2018), *Rdr2* (Hattendorf et al., 2004), *Rdr3* (Whitaker et al., 2009; Zurn et al., 2020), and *Rdr4* (Zurn et al., 2018). In recent years, QTLs for rose black spot resistance that can exist stably in multiple environments or populations have been identified (Yan et al., 2019; Lopez Arias et al., 2020; Rawandoozi et al., 2022; Lau et al., 2023). However, only *Rdr1* has been cloned and functionally validated as an NLR gene (Menz et al., 2018). Therefore, it is crucial to mine the resistance genes for black spot and study the responses of roses to *M. rosae* using other technical means for the selection of black spot-resistant varieties. In recent years, combined analyses of the transcriptome and metabolome have been applied to the study of diseases in multiple species, such as wheat powdery mildew (Xu et al., 2023), broccoli black spot (Shen et al., 2023), peach gummosis (Zhang et al., 2022), and rose powdery mildew (Yang et al., 2022). In a transcription-level study of rose responses to the black spot pathogen, suppression subtractive hybridization (Liu et al., 2015) and massive analysis of cDNA ends (Neu et al., 2019) were used. However, compared with other plant diseases, the omics technology used in the study of rose black spot is relatively backward, it relies on single omics approach and lacks control, and the sampling time is not comprehensive. In addition, few reports have focused on the transcriptomic and metabolic diversity of resistant and susceptible rose varieties to *M. rosae*.

To further explore the diversity of the response to *M. rosae* infection in different resistant rose (*Rosa hybrida*) varieties, this study performed transcriptome and metabolome analyses on leaf samples from resistant and susceptible roses infected with *M. rosae*. The objective of this study was to analyze the genes and metabolites involved in the response to *M. rosae* at the transcriptional and metabolic levels. The results provide a theoretical basis for developing strategies to prevent and control rose black spot and cultivating disease-resistant rose varieties.

## Materials and methods

### Rose materials and *M. rosae* inoculation

The rose (*Rosa hybrida*) varieties used in this study were ‘Princess Michael of Kent’ (KT) and ‘Antique Romantica’ (FG), which showed disease resistance and susceptibility, respectively, after inoculation with the *M. rosae* strain DBE24-1 (Song et al., 2023). Following a previously described inoculation procedure (Song et al., 2023), the leaves of these two varieties were sprayed with conidia of DBE24-1. Additionally, each variety was sprayed with sterile water containing 0.1% Tween-20 as a control. Leaf disease was graded from 0 to 5, with 0 indicating no black spot symptoms and 1, 2, 3, 4, and 5 indicating that the proportion of leaf disease area to total leaf area was 0–1/8, 1/8–1/4, 1/4–1/2, 1/2–3/4, and > 3/4, respectively. The disease index (DI) was calculated according to the method described in a 2019 study (Cheng et al., 2019), and the average of three biological replicates was used to represent the DI for a given time point or treatment. Samples were collected at 0, 1, 3, 6, and 12 days post inoculation (dpi), quickly frozen in liquid nitrogen, and stored in a −80°C freezer. Three biological replicates were sampled per time point per treatment for each variety, and the leaves of each biological replicate were from four to five independent single plants. Fifty-four samples were used for transcriptome sequencing, of which 24 samples collected at 6 and 12 dpi were also used for metabolome analysis. The sample number consisted of the variety (K for KT and F for FG) + treatment (I for inoculation and U for control) - sampling time - biological replicate. For example, KI- 1 d-1 represents the first biological replicate sample collected 1 dpi of KT with *M. rosae*.

### Transcriptome sequencing and expression analysis

Total RNA was extracted from the 54 samples using the RNAprep Pure Plant Kit (Tiangen, Beijing, China). RNA concentration and purity were measured using a NanoDrop 2000 (Thermo Fisher Scientific, Wilmington, DE, USA), and RNA integrity was measured using an Agilent Bioanalyzer 2100 system (Agilent Technologies, CA, USA). After passing the RNA quality inspection, 1 μg per sample was used for the construction of cDNA libraries based on a Hieff NGS Ultima Dual-mode mRNA Library Prep Kit for Illumina (Yeasen Biotechnology (Shanghai) Co., Ltd., Shanghai, China) according to the manufacturer’s instructions. After quality inspection of the library, an Illumina NovaSeq 6000 sequencing platform was used for PE150 mode sequencing to generate 150 bp paired-end reads. cDNA library construction and sequencing were performed by Beijing Biomarker Technologies Co., Ltd. To obtain clean data, Perl was used to remove reads from raw data based on the following criteria: reads containing adapters, reads with a proportion of N greater than 10%, and reads for which the base quality value Q < 10 accounted for more than 50% of the entire read. HISAT2 (Kim et al., 2015) was used to align clean reads to the ‘Old Blush’ reference genome (Raymond et al., 2018) to obtain the physical position information of each read, and only perfectly matched or one-mismatch reads were used for subsequent assembly. The aligned reads were spliced into transcripts using StringTie, new transcripts were discovered, and the transcripts per million (TPM) value was calculated to measure the gene expression levels (Pertea et al., 2015). Principal component analysis (PCA) of the transcriptome samples was performed using the prcomp function in the R. Genes were considered expressed in the sample when their average TPM in biological replicates was greater than 0.05.

### Gene function annotation and differential expression analysis

The protein sequences of the genes were aligned to the NR (Yangyang et al., 2006), Swiss-Prot (Apweiler et al., 2004), and Kyoto Encyclopedia of Genes and Genomes (KEGG) databases (Kanehisa and Goto, 2000), and annotation information was obtained using DIAMOND software (Buchfink et al., 2015). Gene Ontology (GO) annotation (Ashburner et al., 2000) was performed using the InterProScan 5 database (Jones et al., 2014). In addition, HMMER software (Eddy, 1998) was used to align the protein sequences of the genes to the Pfam database (Mistry et al., 2021) to obtain relevant annotation information. The annotation results of the genes in each database were integrated to obtain the functional annotation information of the genes. DESeq2 was used to analyze the differential expression of genes in each comparison (Love et al., 2014). When the fold change was ≥ 2 and the false discovery rate (FDR) was < 0.05, the genes were considered differentially expressed. Both Venn diagrams and heat maps of the differentially expressed genes (DEGs) were drawn using TBtools v2.003 software (Chen et al., 2020). Using the plantTFDB v5.0 database (http://planttfdb.cbi.pku.edu.cn), all species information was selected to predict the transcription factors (TFs) in DEGs and obtain the families to which they belong.

### Clustering and functional enrichment analysis of DEGs

The K-means clustering algorithm of the ComplexHeatmap R package was used to cluster DEGs expression in the FG and KT samples. KEGG and GO enrichment analyses of the DEGs were completed on the BMKCloud platform (www.biocloud.net). In the KEGG enrichment analysis, a p-value < 0.05 for pathway indicated significant enrichment. For the GO enrichment analysis, a q-value < 0.05 for term indicated significant enrichment. A combination plot of gene expression trends, heat maps, and KEGG enrichment results was generated using ClusterGVis (https://github.com/junjunlab/ClusterGVis). GO enrichment bubble plots of DEGs at each time point were generated using https://www.bioinformatics.com.cn/.

### qRT-PCR verification

The RNA samples from the transcriptome described above were reverse transcribed to cDNA using a PrimeScript RT reagent Kit with gDNA Eraser (TaKaRa, RR047A) according to the manufacturer’s instructions. Two WRKY TFs, two AP2/ERF TFs, five pathogenesis-related genes, and one abscisic acid (ABA) receptor were selected from the DEG set. Gene-specific quantitative primers were designed, and UBC and SAND were used as reference genes (Klie and Debener, 2011). The primer sequences are shown in **Supplementary Table 1**. Fluorescence quantitative PCR was performed using ChamQ Universal SYBR qPCR Master Mix (Vazyme Biotech Co. Ltd., China, Q711), with three technical replicates for each biological replicate. Fluorescent signals were detected using a Bio-Rad CFX384 Real-Time System (Bio-Rad, USA). The relative expression of genes was calculated by the 2^-ΔΔCT^ method (Livak and Schmittgen, 2001), and the correlation coefficients between the transcriptome data and qRT-PCR results for each gene were calculated using Microsoft Excel.

### Metabolite detection and differentially accumulated metabolite analysis

Metabolites from 24 samples at 6 and 12 dpi were extracted according to previously described methods (Wang et al., 2016). Metabolite detection was performed using a system combining a Waters ultra-performance liquid chromatograph (UPLC) Acquity I-Class PLUS with a high-resolution mass spectrometer (Waters Xevo G2-XS QTOF). Original data were collected using Waters MassLynx V4.2 software, and peak extraction, alignment, and other data processing were performed using Progenesis QI software. Metabolite identification was performed using the online METLIN database (Smith et al., 2005) and the self-built database from Beijing Biomarker Technologies Co., Ltd. The KEGG (Kanehisa and Goto, 2000), HMDB (Wishart et al., 2018) and LIPID MAPS (Fahy et al., 2007) databases were used to annotate the detected metabolites. Orthogonal projections to latent structures-discriminant analysis (OPLS-DA) were based on sample-to-sample comparisons to obtain the variable importance in projection (VIP) (Thévenot et al., 2015). Metabolites in the two comparisons were considered differentially accumulated metabolites (DAMs) when they satisfied the following criteria: VIP ≥ 1, absolute value of log_2_ (fold change) ≥ 0.585, and P value < 0.05. KEGG enrichment analysis of DAMs was performed using the BMKCloud platform (www.biocloud.net) and KEGG enrichment bubble plots were plotted at https://www.bioinformatics.com.cn/. Correlation analysis of the DAMs and DEGs in each variety at 6 and 12 dpi was performed using TBtools v2.003 (Chen et al., 2020). PCA of the metabolome samples was completed using the prcomp function in the R. In the KEGG pathway, a correlation network diagram of the DAMs and DEGs was plotted using the OmicShare tools (https://www.omicshare.com/tools).

### Phytohormone treatment

24-Epibrassinolide (EBL), one of the most active forms of brassinosteroid, was used to treat rose leaves before inoculation. EBL (Shanghai Yuanye Biotechnology Co., Ltd.) was dissolved in 99.7% ethanol, and a small amount of sterile water was added to prepare a mother liquor of 2 mg/mL. FG leaves were divided into six groups, each containing a total of 6–7 leaves from three individual plants, and sprayed with 0.1, 0.5, 1.5, 3, and 6 mg/L EBL and sterile water (as the control). After 1 d, the DBE24-1-derived conidial solution with reduced pathogenicity was applied by spraying, according to a previously described method (Song et al., 2023). As described above, DI was calculated to measure the extent of disease. Subsequently, GraphPad Prism 8 was used to perform one-way ANOVA for the DI between groups and plot a histogram.

## Results

### Resistant and susceptible roses showed significant differences after inoculation with *M. rosae*

**Figure 1:**
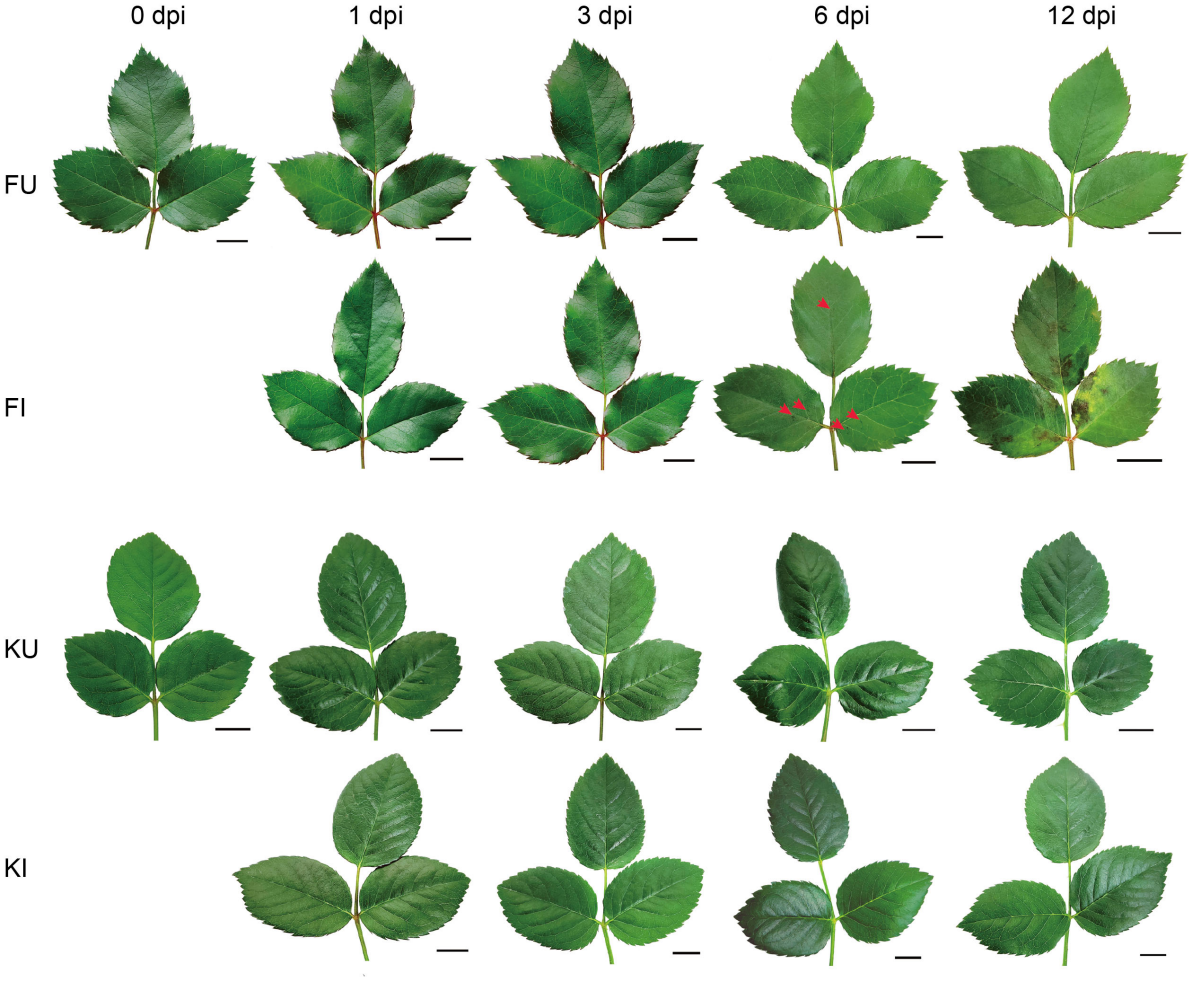
FG and KT phenotypes after inoculation with *M. rosae*. FI and KI represent the treatments of FG and KT sprayed with a conidial solution of *M. rosae*, respectively. FU and KU represent the treatments of FG and KT sprayed with sterile water as controls, respectively. Red arrows indicate initial symptoms, and the bar is 1 cm.

Previously, the pathogenic strain DBE24-1 was isolated from rose black spot leaves (Song et al., 2023) and inoculated into the leaves of FG and KT plants, which showed susceptibility and resistance, respectively. At 1 and 3 dpi, no symptoms were observed in the leaves of either FT or KT (DI=0). Punctate disease spots appeared on the leaves of the susceptible variety at 6 dpi (**Figure 1**), with a DI of 17.04 (**Supplementary Figure 1A**). The resistant variety did not show any disease symptoms until 12 dpi (DI=0), whereas the susceptible variety showed large disease spots on the leaves and local yellow areas around the disease spots at 12 dpi (**Figure 1**) (DI=50.37) (**Supplementary Figure 1A**). The controls of the two varieties that were not inoculated with *M. rosae* did not exhibit pathogenic symptoms (**Figure 1**). These results indicate a significant difference in the phenotypes of the two varieties after inoculation with *M. rosae*, and it is speculated that they responded differently to *M. rosae*.

### Quality control and alignment analysis of transcriptome data

**Figure 2:**
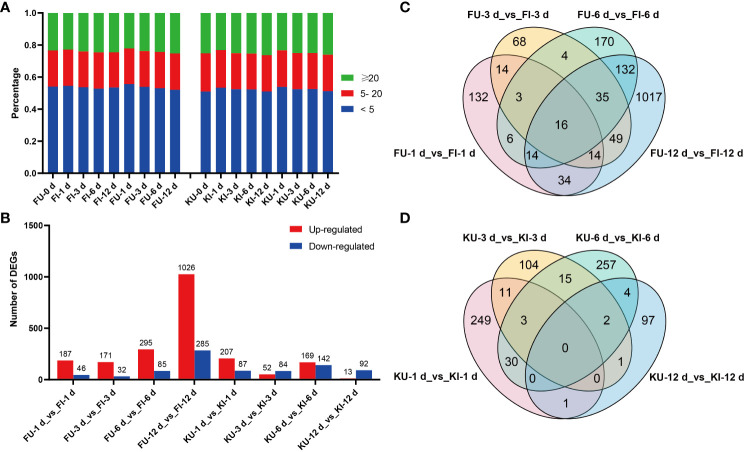
Gene expression and differential expression analyses of FG and KT samples. **(A)** Percentage of each sample in the different TPM ranges. **(B)** Number of differentially expressed genes (DEGs) that were upregulated and downregulated in each comparison. Venn plots of DEGs in the four comparisons for FG **(C)** and KT **(D)**.

Fifty-four transcriptome sequencing samples were filtered to obtain a total of 358.88 Gb of clean data, and the average clean data of a single sample was 6.65 Gb. The average GC content of the samples was 45.97%, average Q20 value was 98.28%, and average Q30 value ranged from 91.62–95.46%. This showed that the accuracy and quality of the sequenced reads were sufficiently high to meet the needs of subsequent analyses. In the FG and KT samples, the average alignment rates of clean reads on the genome were 85.58% and 84.50%, respectively, and the unique alignment rates of clean reads were 82.02% and 81.36%, respectively (**Supplementary Table 2**). The distribution of TPM values was relatively consistent across samples, with more than half of the genes at TPM < 5 and a low number of genes at TPM ≥ 20 (**Figure 2A**). Based on the reference genome sequence, 2822 new genes (gene number prefix NewGene) with annotated information were obtained using StringTie software to splice the mapped reads (**Supplementary Table 3**). Correlation analysis between the samples showed that the three biological replicates presented correlation coefficients of 0.8752–0.9996. However, a few samples (KI-3 d-1, KI-12 d-3, and KU-12 d-3) presented higher correlations between groups than within groups (**Supplementary Table 4**). Therefore, these three samples were removed and the remaining samples were used for subsequent analyses. PCA was performed on the transcriptome samples, and it was found that the sample distribution in FG and KT were significantly different; the sample could be divided into two groups according to the variety. Notably, in both varieties, the distributions of the samples collected at 0 and 1 dpi were markedly distant from those of the samples collected at other time points (**Supplementary Figure 2**).

### Involvement of multiple TFs in rose responses to *M. rosae*

To study the transcriptional level changes of genes in FG and KT during *M. rosae* infection, differential expression analysis was performed on the inoculated and uninoculated samples at four time points for the two varieties (FU vs. FI and KU vs. KI). FG had more upregulated genes than downregulated genes at the four time points, with a minimum of 203 DEGs at 3 dpi and a maximum of 1311 DEGs at 12 dpi (**Figure 2B**). FG contained 1708 genes that were differentially expressed during at least one time point (**Figure 2C**). Among the four comparisons in KT, the number of upregulated DEGs was higher than that of downregulated DEGs at 1 and 6 dpi, with a minimum of 105 DEGs at 12 dpi and a maximum of 311 DEGs at 6 dpi (**Figure 2B**). KT had 774 genes that were differentially expressed during at least one time point (**Figure 2D**). Sixteen DEGs were common to FG at the four time points (**Figure 2C**). Among them, 15 genes were upregulated at the four time points, and the other was downregulated. According to the annotation results, at least seven of these genes encode pathogen-related proteins (**Supplementary Figures 1B, C**). There were no common DEGs at the four time points in KT (**Figure 2D**).

TFs regulate gene expression by binding specifically to promoter regions. The plantTFDB database was used to identify TFs responses to *M. rosae* in FG and KT. A total of 102 TFs were found to respond to *M. rosae* (RM-TFs) in FG. They belonged to 21 TF families, among which WRKY (24), AP2/ERF (19), MYB (9), and NAC (8) were the most abundant (**Supplementary Figure 3A**, **Supplementary Table 5**). Compared with FU, more than half of the RM-TFs in FI were upregulated, especially at 12 dpi (**Supplementary Figures 4A, C**). There were 28 RM-TFs in KT, and they belonged to 14 TF families, among which AP2/ERF (8), C2H2 (5), and WRKY (3) were the most abundant (**Supplementary Figure 3B**, **Supplementary Table 5**). In KT, RM-TFs were highly expressed in the KU-12 d samples (**Supplementary Figure 4B, C**), which was different from those in FG. The two varieties had eight RM-TFs in common, and their expression patterns differed between the varieties, with six showing large variation in expression levels in FG (**Supplementary Figure 4C**). This indicates that there were differences in the number and expression patterns of RM-TFs between FG and KT, which may cause downstream gene expression changes.

### Diversity of GO term enrichment within the DEGs of resistant and susceptible roses

**Figure 3:**
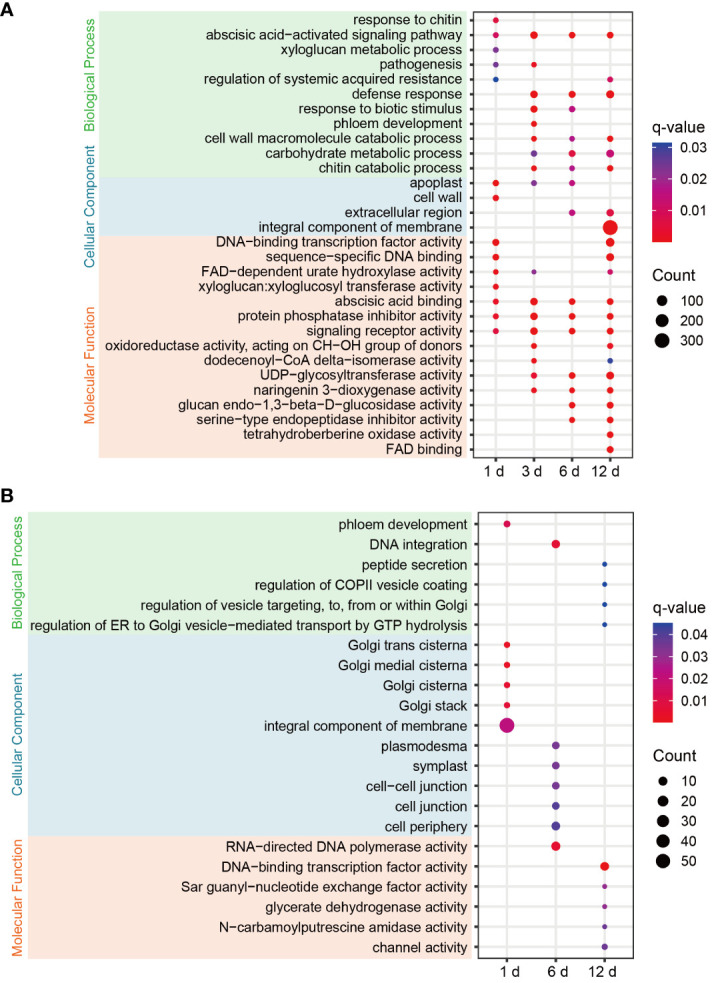
GO enrichment bubble plots of DEGs for each comparison in FG **(A)** and KT **(B)**. Only significantly enriched terms are shown (q-value < 0.05). The color of the circle from blue to red indicates the q-value from large to small, and the size of the circle indicates the number of genes enriched in the term.

To clarify the functional characteristics and differences of DEGs in FG and KT, GO enrichment analysis was performed on the DEGs at the four time points for the two varieties. FG was significantly enriched in more terms than KT at each time point (**Figure 3**). Among the most significantly enriched terms in FG, the “abscisic acid-activated signaling pathway” in the biological process category was significantly enriched at four time points. The “defense response,” “cell wall macromolecule catabolic process,” “carbohydrate metabolic process,” “chitin catabolic process,” “response to biotic stimulus,” and “pathogenesis” terms were also significantly enriched during at least two time points, and they were directly or indirectly related to plant defense response to pathogen. In the cellular component category, “apoplast” was significantly enriched at the first three time points, “extracellular region” was significantly enriched during the last two time points, and “cell wall,” and “integral component of membrane” were significantly enriched at 1 and 12 dpi, respectively (**Figure 3A**). The cell wall and apoplast are the main locations where plants and microbes interact (Dora et al., 2022). In the molecular function category, “abscisic acid binding,” “protein phosphatase inhibitor activity,” and “signaling receptor activity” were significantly enriched at all time points. Among them, “abscisic acid binding” seems to correspond to the significant enrichment of the “abscisic acid-activated signaling pathway.” “Xyloglucan:xyloglucosyl transferase activity” was significantly enriched at 1 dpi (**Figure 3A**). Xyloglucan:xyloglucosyl transferase is extremely important for cell-wall remodeling during plant responses to biotic stress (Hrmova et al., 2022), and it corresponded to “cell wall” enrichment at 1 dpi. In summary, the response of FG to *M. rosae* involves the abscisic acid (ABA) signaling pathway, cell wall, defense response, and other related biological processes. The distribution characteristics of the enriched terms at the four time points appear to be related to the process of *M. rosae* invading plant cells.

Significantly enriched terms were screened based on a q-value < 0.05. DEGs were not significantly enriched in any of the terms at 3 dpi in KT. Among the significantly enriched terms for DEGs at the other three time points, “phloem development” and “DNA integration” in the biological process category were significantly enriched at 1 and 6 dpi, respectively. The following terms related to vesicle trafficking were significantly enriched at 12 dpi: “peptide secretion,” “regulation of COPII vesicle coating,” “regulation of vesicle targeting, to, from or within Golgi,” and “regulation of ER to Golgi vesicle−mediated transport by GTP hydrolysis.” In the cellular component category, DEGs at 1 dpi were significantly enriched in Golgi-related molecular components, such as “Golgi trans cisterna,” “Golgi medial cisterna,” “Golgi cisterna,” and “Golgi stack.” DEGs at 6 dpi were significantly enriched in “plasmodesma,” “symplast,” “cell−cell junction,” “cell junction,” and “cell periphery.” In the molecular function category, DEGs at 12 hpi were significantly enriched in “DNA-binding TF activity,” “Sar guanyl-nucleotide exchange factor activity,” “glycerate dehydrogenase activity,” “N-carbamoylputrescine amidase activity,” “channel activity,” and other terms (**Figure 3B**). In summary, the response of KT to *M. rosae* might be related to Golgi-mediated vesicle trafficking.

### Significant differences in DEGs involved in pathogen response pathways in resistant and susceptible roses

**Figure 4:**
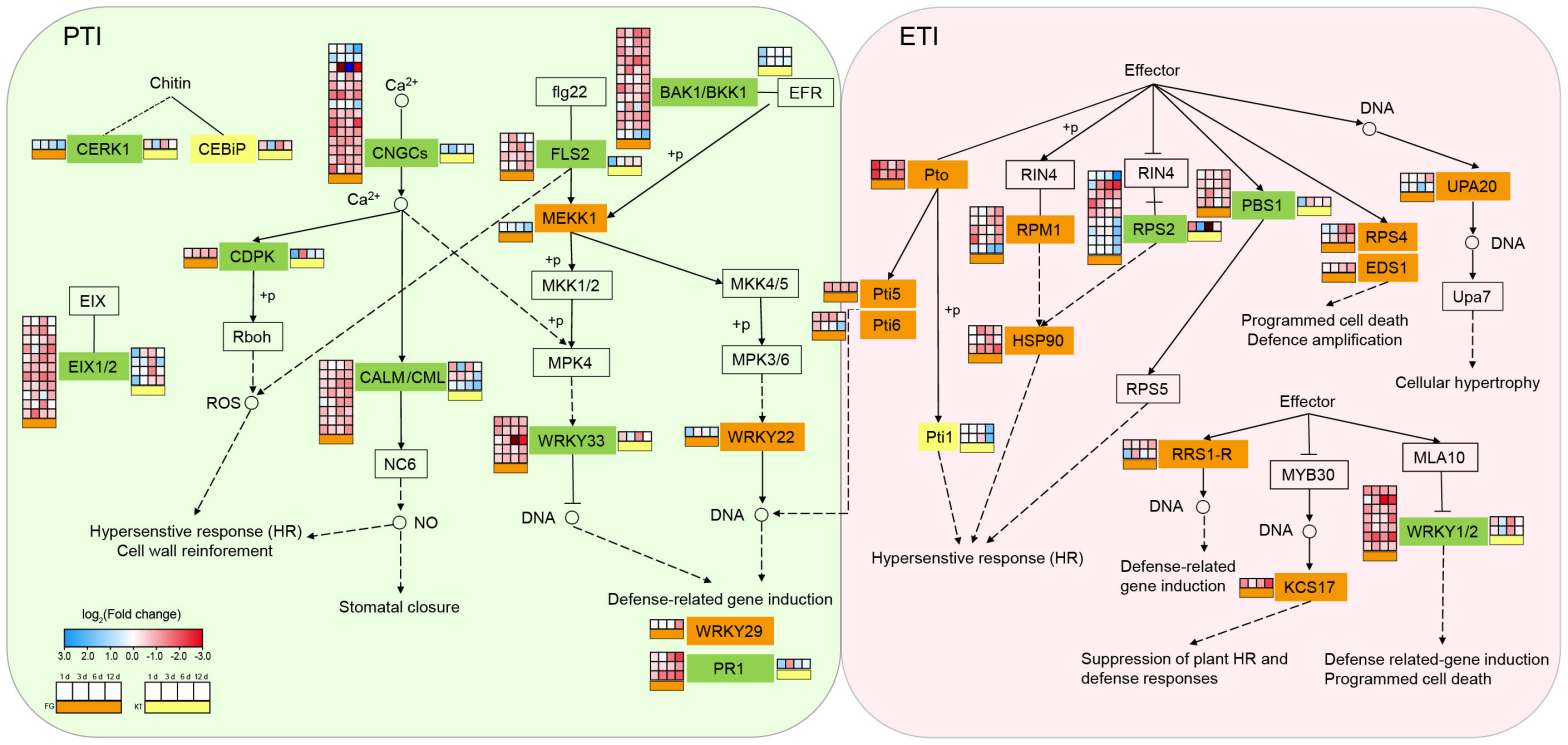
Changes in the expression of DEGs in the plant–pathogen interaction pathway for FG and KT. The gene names at various locations along the pathway are contained within rectangles. Different colors are used to fill the rectangles to distinguish the types of DEGs. Rectangles in the background color with a black border, indicate that there are no DEGs in either of the two varieties. Orange, yellow, and green rectangles represent the presence of DEGs only in FG, only in KT, and in both FG and KT, respectively. The four-column squares from left to right represent the log_2_ values of the TPM ratio of inoculated versus uninoculated samples at 1, 3, 6, and 12 dpi. The orange bars under the squares indicate comparisons for FG (placed to the left of the gene name), and the yellow bars indicate comparisons for KT (placed to the right of the gene name). Red and blue indicate upregulated and downregulated DEGs, respectively.

To study the biological pathways involved in DEGs, KEGG classification was performed on the DEG sets of the two varieties separately. Among the pathways common to the two varieties, the largest number of DEGs belonged to the “plant–pathogen interaction,” “plant hormone signal transduction,” and “MAPK signaling pathway-plant” pathways (**Supplementary Figure 5**). Therefore, these three pathways generally considered to be related to pathogenic responses were further analyzed. The plant–pathogen interaction pathway encompasses two aspects of plant defense against pathogens: PTI and ETI. These two immune systems were activated in both the resistant and susceptible varieties after inoculation. The numbers of DEGs belonging to this pathway in FG and KT were 103 and 22, respectively (**Supplementary Figure 5**, **Supplementary Table 6**). One CEBiP gene (RcHm_v2.0_Chr6g0280231) involved in PTI and two Pti1 genes (RcHm_v2.0_Chr7g0223691 and RcHm_v2.0_Chr7g0226681) involved in ETI were unique DEGs in KT. The unique DEGs in FG were mainly concentrated in ETI, except for MEKK1 (NewGene_15861), WRKY22 (RcHm_v2.0_Chr1g0378621), and WRKY29 (RcHm_v2.0_Chr7g0196571), which were involved in PTI (**Figure 4**; **Supplementary Table 6**). These results indicated that the immune response elicited by inoculation with *M. rosae* was more intense in FG than in KT.

**Figure 5:**
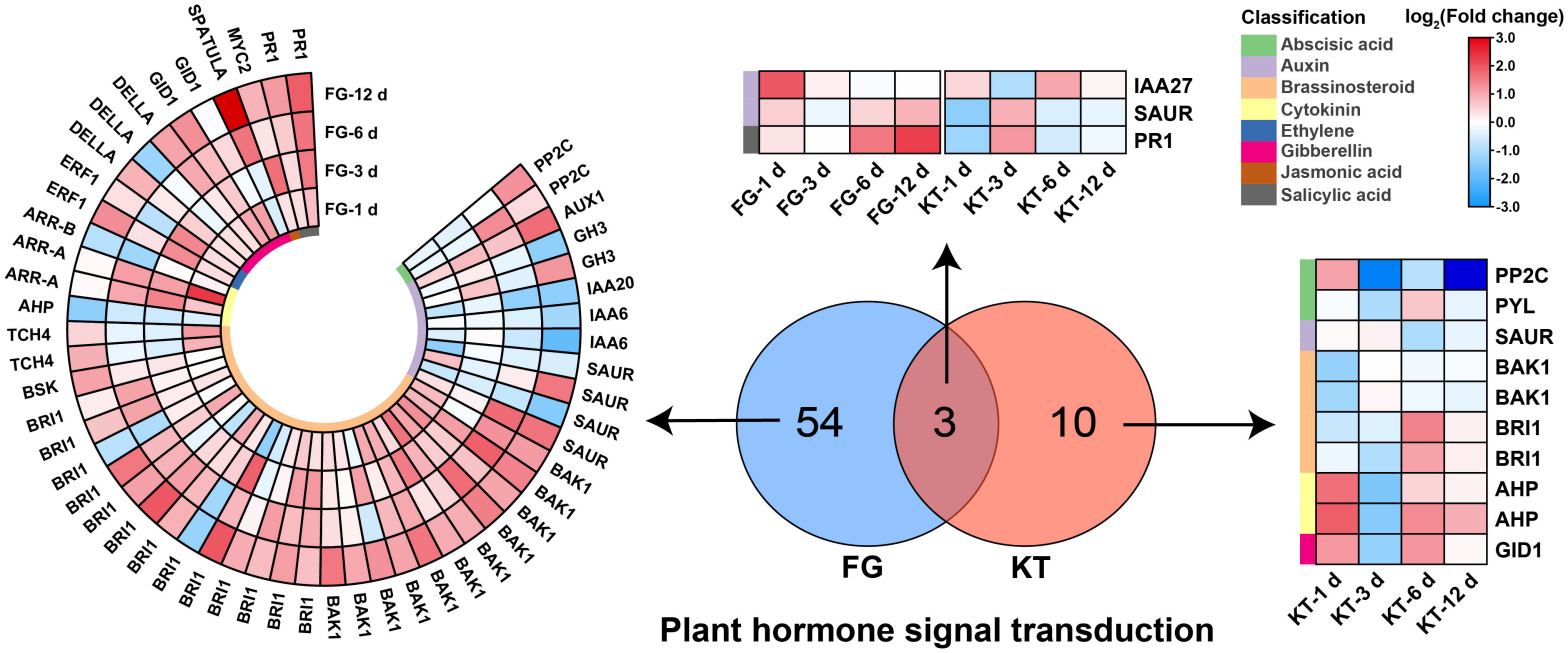
Heat maps of DEGs derived from the plant hormone signal transduction pathway in FG and KT. The colored blocks on the left side of the heat map indicate different hormones, and the upregulation and downregulation of the DEGs are indicated in red and blue, respectively.

A total of 57 DEGs belonging to “plant hormone signal transduction” were found in FG; they included eight hormones: ABA, auxin, BR, cytokinin, ethylene (ET), gibberellin, jasmonic acid (JA), and salicylic acid (SA). The largest number of DEGs belonged to the BR signal transduction pathway (27), which were mainly upregulated, followed by the auxin signal transduction pathway (12), which were mainly downregulated (**Figure 5**; **Supplementary Table 7**). Only 13 DEGs belonged to the plant hormone signal transduction pathway in KT, and they were associated with six hormone signal transduction pathways, excluding ET and JA. There were three common DEGs between KT and FG in this pathway, and they were mainly upregulated in FG (**Figure 5**; **Supplementary Table 7**). In summary, the responses of roses to *M. rosae* involved a variety of plant hormone signal transduction pathways, and the number and types of DEGs were significantly different between FG and KT.

A greater number of DEGs belonging to the “MAPK signaling pathway-plant” was found in FG (43) than in KT (10). The common DEGs were WRKY33 (RcHm_v2.0_Chr3g0485711) and PR1 (RcHm_v2.0_Chr6g0247671), which were mainly upregulated in FG. The 41 DEGs unique to FG were also mainly upregulated, while the eight DEGs unique to KT were mainly downregulated (**Supplementary Figure 6**, **Supplementary Table 8**). In general, the resistant and susceptible varieties responded to *M. rosae* by reducing and increasing the expression of genes in this pathway, respectively.

In addition, DEGs in FG were also involved in the biosynthesis and metabolism of a variety of metabolites and amino acids, such as “phenylpropanoid biosynthesis” (49), “starch and sucrose metabolism” (32), “cutin, suberine and wax biosynthesis” (18), etc. (**Supplementary Figure 5A**). The same was true in KT, where there were more DEGs belonging to “starch and sucrose metabolism” (10), “valine, leucine and isoleucine degradation” (9), and “galactose metabolism” (8) (**Supplementary Figure 5B**). In general, DEGs were involved in multiple biological pathways, indicating that the response of rose leaves to *M. rosae* is not a simple single linear cascade reaction but rather a complex combination of multiple signal transduction pathways.

### Temporal expression trends of DEGs showed diversity in resistant and susceptible roses

**Figure 6:**
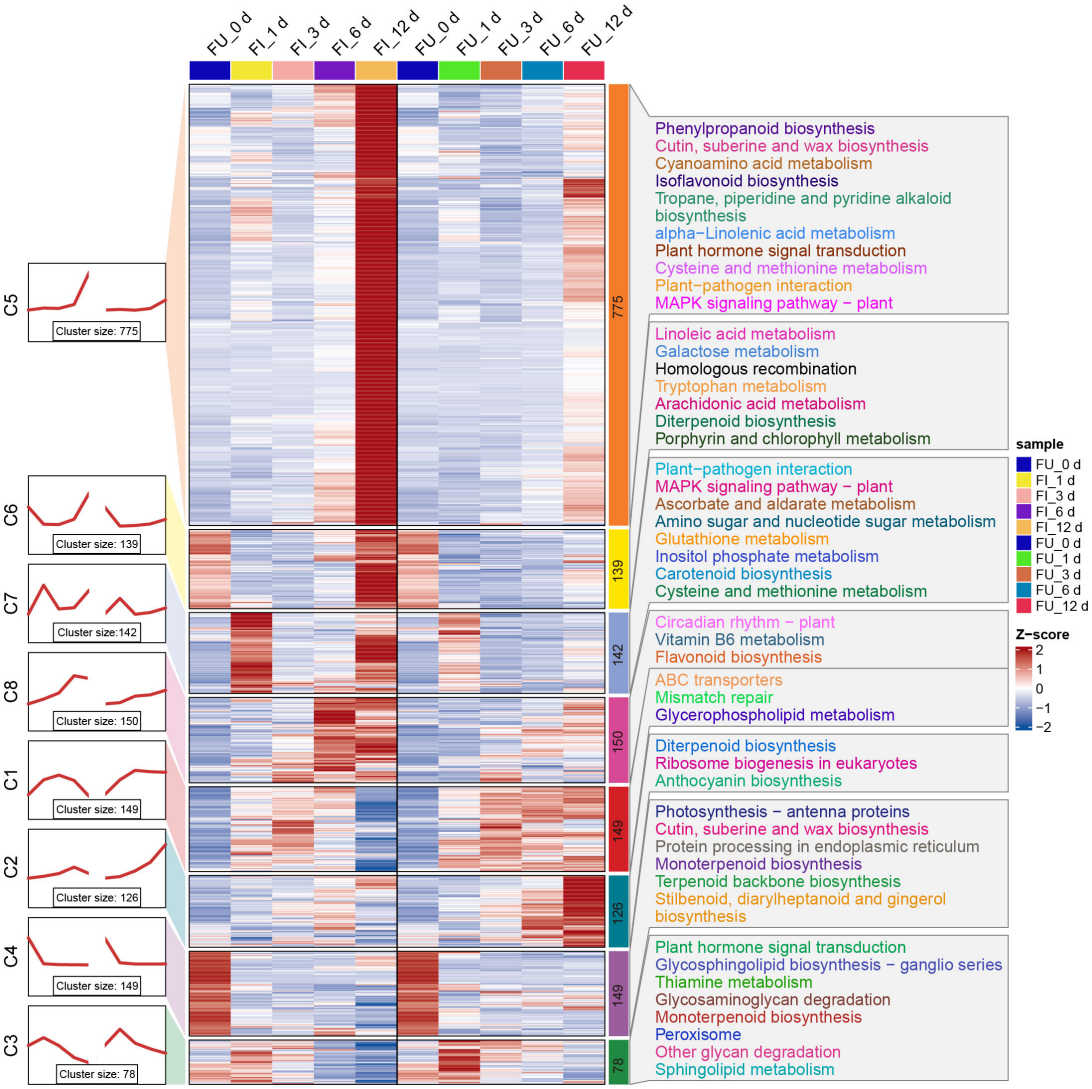
Expression trends, heat maps, and significantly enriched KEGG pathways of each cluster of DEGs in FG. The left side shows a line plot of the expression trend of DEGs in each cluster of the FI and FU samples. The left side of the rectangle shows the serial number of clusters, and the number of genes within each cluster (cluster size) is shown below the line plot. In the middle, the heat maps of each cluster of DEGs in the FI and FU samples are shown, and changes in the color of the heat map from blue to red indicate changes in expression level from low to high. The right side shows the pathways that were significantly enriched for DEGs in each cluster after KEGG enrichment analysis.

As described above, 1708 and 774 DEGs were observed for FG and KT, respectively, during at least one time point (**Figures 2C, D**). To further study the temporal expression changes of DEGs, K-means clustering analysis was performed on these two DEG sets, and it showed that DEGs with similar expression patterns clustered into one cluster. Then a KEGG enrichment analysis was performed on the DEGs of each cluster. In FG, DEGs were clustered into eight clusters, each containing 78–775 genes. In clusters 5, 6, 7, and 8, the expression of genes in FI was relatively higher than that in FU, whereas in clusters 1, 2, and 3, the opposite was true (**Figure 6**). Overall, the expression pattern of DEGs in each cluster was similar in the FI and FU samples, although both the gene expression pattern and amplitude varied more in FI. DEGs in clusters 5 and 7 were significantly enriched in the “plant-pathogen interaction” and “MAPK signaling pathway-plant” and mainly upregulated at 1 and 12 dpi (**Figure 6**). DEGs in clusters 3 and 5 were significantly enriched in the “plant hormone signal transduction,” although the expression patterns of DEGs in the two clusters were quite different (**Figure 6**). The expression pattern of DEGs in cluster 5 from FI was similar to the line chart of the DI for FG (**Supplementary Figure 1A**).

The 774 DEGs in KT were divided into eight clusters, with the largest (cluster 4) and smallest (cluster 3) clusters containing 164 and 59 genes, respectively. Compared with FG, the expression patterns of KI and KU in KT were less similar; in addition, the expression patterns of genes in the KI clusters were more complex (**Supplementary Figure 7**). KEGG enrichment analysis was performed on the DEGs of the eight clusters, which showed that clusters 3, 7, and 8 were significantly enriched in “anthocyanin biosynthesis.” However, the expression pattern of cluster 3 was quite different from that of the other two clusters. Clusters 4, 5, and 6 were significantly enriched in “galactose metabolism,” and clusters 4 and 5 were significantly enriched in “ascorbate and aldarate metabolism” (**Supplementary Figure 7**). Genes belonging to the same pathway participated in the response to *M. rosae* through different expression patterns in KT. It is worth noting that the expression level of DEGs in clusters 5 showed a continuous upward trend in the early and middle stages of pathogen infection (**Supplementary Figure 7**). Moreover, the pathways enriched in these DEGs, specifically lysine degradation, flavonoid biosynthesis, and phenylpropanoid biosynthesis, were reported to be associated with plant disease resistance (Shinde et al., 2017; Hartmann and Zeier, 2018; Li et al., 2021).

### Validation of transcriptome results via qRT-PCR

To verify the reliability of the transcriptome results, two WRKY TFs, two AP2/ERF TFs, five pathogenesis-related genes, and one ABA receptor were selected from the DEGs for qRT-PCR. The housekeeping genes UBC and SAND were used as internal controls. The relative expression of each gene was calculated and plotted together with the average TPM values of the samples in the transcriptome. The expression trend of the genes determined by qRT-PCR was consistent with the transcriptome results. Correlation analysis of the FG and KT results showed that the average correlation coefficients were 0.966 and 0.834, respectively (**Supplementary Figure 8**), which further indicated that the data obtained by transcriptome sequencing were reliable.

### Diverse metabolomic profiles in response to *M. rosae* infection in resistant and susceptible roses

To further identify the metabolites in FG and KT that respond to *M. rosae* infection, 24 samples collected at 6 and 12 dpi were selected for non-targeted metabolite detection. In the mode without distinguishing between positive and negative ions, 14,365 peaks were detected and 3,731 metabolites were annotated. The classes with the highest number of metabolites were organooxygen compounds (212), prenol lipids (206), carboxylic acids and derivatives (197), and fatty acyls (174) (**Supplementary Figure 9A**). Correlation analysis of the samples showed that the correlation coefficients between the biological replicates of each group were between 0.985 and 0.995 (**Supplementary Figure 10**), which met the requirements of the follow-up analysis. PCA of the metabolome samples revealed a discernible demarcation in the distribution of samples between the two varieties, with the three biological replicates of each sample demonstrating substantial clustering. The distribution of FG samples at both 6 and 12 dpi was more scattered than that of KT samples. This dispersion may be attributed to the significant phenotypic differentiation between the inoculated and non-inoculated FG samples at these respective time points (**Supplementary Figure 11**). The difference comparison analysis (U vs. I) at each time point in the two varieties showed that the number of upregulated DAMs was higher than that of downregulated DAMs in each comparison (**Supplementary Figure 9B**), indicating that the two varieties mainly responded to the invasion of *M. rosae* by increasing metabolites. The number of DAMs in FG was significantly higher than that in KT, which was consistent with the finding that the number of DEGs in FG was higher than that in KT. Thus, metabolite changes in the resistant variety were more stable than those in the susceptible variety after infection.

**Figure 7:**
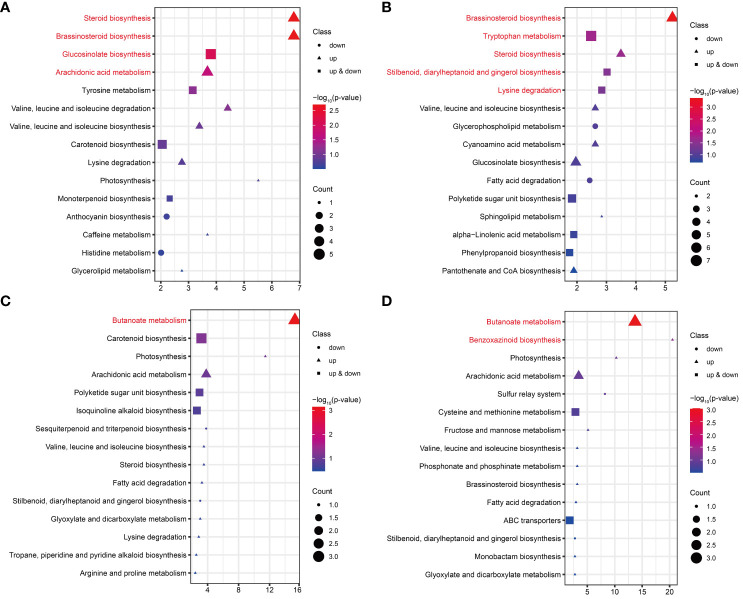
KEGG enrichment analysis of DAMs in FG and KT for each comparison. Top 15 pathways for KEGG enrichment of DAMs at 6 dpi **(A)** and 12 dpi **(B)** in FG. Top 15 pathways for KEGG enrichment of DAMs at 6 dpi **(C)** and 12 dpi **(D)** in KT. The pathways with significant enrichment are shown in red. Genes that were upregulated, downregulated, and both upregulated and downregulated in the given pathway are represented by triangles, circles, and squares, respectively. The size of these three shapes indicates the number of DAMs in the pathway, and the colors from red to blue indicate the -log_10_ (p-value) from large to small.

KEGG enrichment analysis was performed on the DAMs for each comparison in FG and KT. The significant enrichment pathways of DAMs from FU-6d_vs_FI-6d included the “steroid biosynthesis,” “brassinosteroid biosynthesis,” “glucosinolate biosynthesis,” and “arachidonic acid metabolism.” Although the DAMs associated with the “glucosinolate biosynthesis” pathway were both upregulated and downregulated, the DAMs in the rest of the pathways were only upregulated (**Figure 7A**). The DAMs in FU-12d_vs_FI-12d were also significantly enriched in the “brassinosteroid biosynthesis” and “steroid biosynthesis”, and these DAMs also showed upregulation accumulation. In addition, the “tryptophan metabolism,” “stilbenoid, diarylheptanoid and gingerol biosynthesis,” and “lysine degradation” were also significantly enriched (**Figure 7B**). The DAMs in KU-6d_vs_KI-6d and KU-12d_vs_KI-12d were significantly enriched in the butanoate metabolism pathway, while the DAMs in KU-12d_vs_KI-12d were significantly enriched in the benzoxazinoid biosynthesis pathway (**Figures 7C, D**). The above results show that FG and KT present obvious differences in the biological pathways that respond to *M. rosae*, although both mainly increased the accumulation of metabolites.

### Potential negative regulation of black spot resistance by brassinosteroid biosynthesis in susceptible rose

We performed an integrated analysis of the metabolomes and transcriptomes in response to *M. rosae* infection. The DAMs at 6 and 12 dpi in the two varieties were combined, and the numbers of DAMs that occurred at least at one time point in FG and KT were 378 and 154, respectively (**Supplementary Figure 9C**). After merging the DEGs of each variety at the two time points, there were 1494 DEGs in FG and 411 DEGs in KT (**Figures 2C, D**). KEGG enrichment analysis was performed on these DAMs and DEGs, and 49 (**Supplementary Table 9**) and 15 (**Supplementary Table 10**) co-enrichment pathways of DAMs and DEGs were observed in FG and KT, respectively.

**Figure 8:**
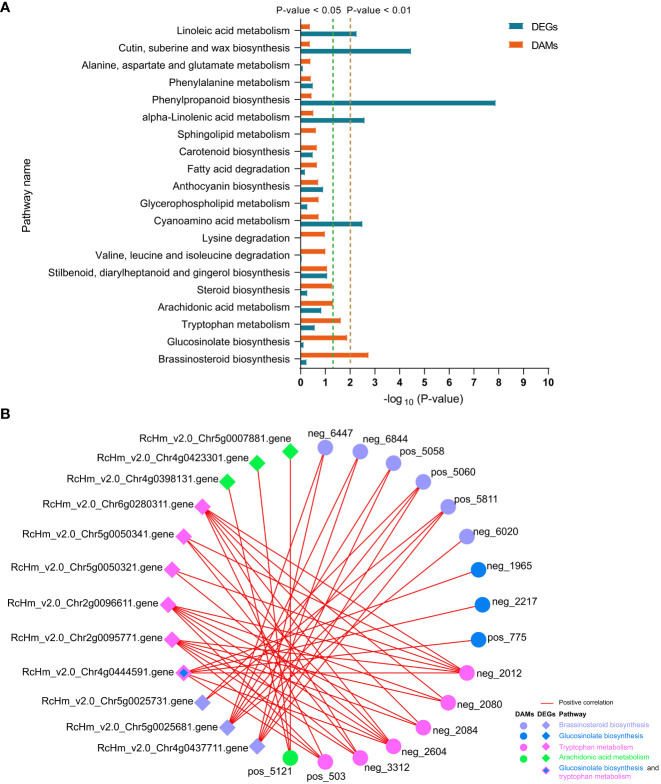
Top 20 co-enriched KEGG pathways in DEGs and DAMs at 6 and 12 dpi in FG **(A)**, co-expression networks of DAMs and DEGs in the four significantly enriched pathways **(B)**. Red lines indicate positive correlations between DAMs and DEGs, and circles and diamonds indicate DAMs and DEGs, respectively. Different colors are used to distinguish the pathways to which the DAMs and DEGs belong.

In FG, DAMs were significantly enriched in the “brassinosteroid biosynthesis,” “glucosinolate biosynthesis,” “tryptophan metabolism,” and “arachidonic acid metabolism” pathways (**Figure 8A**). Correlation analysis revealed that DAMs and DEGs in the four pathways were significantly positively correlated in FG. Six metabolites from the BR biosynthesis pathway, neg_6447 [(22R,23R)-22, 23-dihydroxycampesterol], neg_6844 (teasterone), pos_5058 (castasterone), pos_5060 (6-Deoxocathasterone), pos_5811 (22alpha-Hydroxy-campesterol), and neg_6020 (brassinolide), showed highly significant positive correlations with RcHm_v2.0_Chr5g0025681 (**Figure 8B**; **Supplementary Table 11**). The gene encodes cytochrome P450 CYP749A22-like, which is involved in BR biosynthesis (**Supplementary Table 11**). In the glucosinolate biosynthesis pathway, neg_1965 (S-(phenylacetothiohydroximoyl)-L-cysteine), neg_2217 (3-Methyl-2-oxobutanoic acid), and pos_775 (L-valine) showed a significant positive correlation with the gene encoding UDP-glycosyltransferase 74B1-like (RcHm_v2.0_Chr4g0444591). In addition, a significant positive correlation was observed between this gene and the three metabolites of the tryptophan metabolism pathway (**Figure 8B**; **Supplementary Table 11**). These two pathways appear to be linked to the tryptophan biosynthesis pathway as mediators. In the tryptophan metabolism pathway, six DAMs were positively correlated with six DEGs to varying degrees. Among these, neg_2012 and neg_2604 were significantly positively correlated with six DEGs. RcHm_v2.0_Chr2g0095771, RcHm_v2.0_Chr2g0096611, and RcHm_v2.0_Chr6g0280311 were significantly positively correlated with all six DAMs (**Figure 8B**, **Supplementary Table 11**). Among these, RcHm_v2.0_Chr2g0095771 and RcHm_v2.0_Chr6g0280311 are members of the YUCCA gene family (**Supplementary Table 11**) and mediate the tryptophan-dependent auxin synthesis pathway (Solanki and Shukla, 2023).

**Figure 9:**
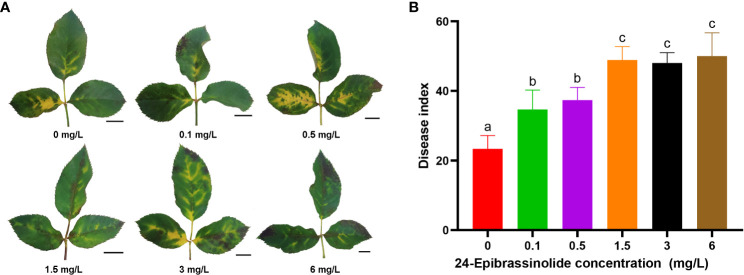
Disease phenotypes after treatment of FG leaves with different concentrations of 24-epibrassinolide (EBL). Typical morphology of FG leaves after treatment with EBL at each concentration **(A)**. Histogram of the FG disease index after EBL treatment at six concentrations **(B)**. The letters a, b, and c were used to mark the significance of multiple comparisons. Different letters at the top of two treatments indicate significant differences (P < 0.05).

As described above, the number of DEGs belonging to the BR signaling pathway was found to be the largest among the eight hormones in FG (**Figure 5**). Therefore, FG leaves were treated with five concentrations of EBL for 24 h and then sprayed with a conidial solution of *M. rosae*. The results showed that the disease degree of the FG leaves was aggravated after treatment with all five concentrations of EBL compared with that of the control. The DI of each treatment was significantly higher than that of the control (**Figure 9**).

Among the 15 co-enriched pathways for DAMs and DEGs in KT, only the “anthocyanin biosynthesis” and “amino sugar and nucleotide sugar metabolism” were significantly enriched for DEGs (**Supplementary Figure 12A**). However, significant correlations were not observed between the DAMs and DEGs belonging to these two pathways. No pathways were significantly enriched for DAMs in KT, and among the five pathways with the smallest p-values, pos_1330, which only belonged to the “sesquiterpenoid and triterpenoid biosynthesis,” showed a highly significant positive correlation with RcHm_v2.0_Chr6g0305391 (**Supplementary Figure 12B**). This indicated that the co-expression relationship between DAMs and DEGs was stronger in FG than in KT.

## Discussion

*M. rosae* is a hemibiotrophic ascomycetous fungus that is usually identified at the asexual stage and rarely identified at the sexual phase (*D. rosae*). The biotrophic stage uses haustoria as organs for absorbing nutrients from host plants. At approximately 6 dpi, the leaves of susceptible species form necrotrophic intracellular hyphae that grow between the host cells and enter the necrotrophic stage, followed by the production of new conidia (Gachomo et al., 2006; Gachomo and Kotchoni, 2007). *M. rosae* has a long period from spore germination to the production of new conidia on rose leaves, and its black spot phenotype is usually investigated from 10 to 14 dpi (von Malek and Debener, 1998; Zurn et al., 2018), which is longer than typical investigations in other diseases (Gao et al., 2021; Zhang et al., 2022).

Liu et al. (2015) studied the early response of a resistant variety to *M. rosae* by constructing a suppression subtractive hybridization library, and the analyzed inoculation and control libraries were composed of equal amounts of RNA mixed for 1, 2, and 3 dpi (Liu et al., 2015). Neu et al. (2019) used massive analysis of cDNA ends to analyze changes in the transcription levels of genes caused by the affinity interaction between the susceptible variety and *M. rosae* at the early stages (0, 1, and 3 dpi) (Neu et al., 2019). At present, studies on the transcription levels of roses in response to *M. rosae* have mainly focused on the early stages (3 dpi and earlier), while few studies have investigated the transition from the biotrophic stage to the necrotrophic and late necrotrophic stages. Compared to other rose diseases (Gao et al., 2021; Yang et al., 2022), the omics technology used in the study of black spot disease is relatively outdated. In addition, few studies have compared the responses of resistant and susceptible roses to *M. rosae* under the same conditions. In this study, transcriptome sequencing analysis of resistant and susceptible roses inoculated with *M. rosae* was performed at five different time points. These time points included 0, 1, and 3 dpi during the biotrophic stage, 6 dpi during the trophic mode transition, and 12 dpi at the necrotrophic stage for phenotypic investigation. Compared with previous studies, the sampling stage in this study was more comprehensive and the responses of resistant and susceptible roses to *M. rosae* were compared at the transcriptional and metabolic levels.

Neu et al. (2019) studied the early response of a susceptible variety to *M. rosae* and found that DEGs were enriched in GO terms, such as “chitin catabolic process,” “defense response,” and “response to biotic stimulus” (Neu et al., 2019). In the present study, DEGs from the early stages of FG were also enriched in these GO terms, indicating that these terms may be common to susceptible roses in response to *M. rosae*. In addition, the response of FG to *M. rosae* involved related GO terms such as cell wall and ABA signaling (**Figure 3**). To successfully infect plants, pathogens must destroy the structure of the cell wall by secreting cell wall-degrading enzymes. Substances produced after cell wall degradation can activate immune responses in plants (De Lorenzo et al., 2018). The expression analysis of DEGs within the plant-pathogen interaction pathway indicated that the immune response was activated in FG but was not sufficient to resist the invasion of *M. rosae*, thus leading to susceptibility (**Figure 1**). Similar to the results of Neu et al (Neu et al., 2019), several pathogenesis-related genes and WRKY TFs were upregulated in FG (**Supplementary Figure 8**). Liu et al. (2015) studied the early response of a resistant rose variety to *M. rosae* and performed a GO functional analysis, which revealed that DEGs enriched in reactive oxygen removal, the photorespiration pathway, and the phenylpropanoid metabolism pathway may play an important role in resistance to *M. rosae* infection (Liu et al., 2015). However, in this study, the DEGs of KT in response to *M. rosae* in the early stage were mainly enriched in Golgi-related components, such as the “Golgi trans cisterna,” “Golgi medial cisterna,” “Golgi cisterna,” and “Golgi stack.” Additionally, the DEGs were significantly enriched in peptide secretion and biological processes associated with Golgi-mediated vesicle trafficking during the necrotrophic stage (**Figure 3**). Vesicle trafficking plays an important role in plant defense responses. Plant cells can use vesicles to transport pathogenesis-related substances, such as proteins, nucleic acids, lipids, and metabolites, to pathogen infection sites to prevent pathogen invasion (Abubakar et al., 2023). Therefore, it is speculated that the resistance of KT to *M. rosae* may be related to Golgi-mediated vesicle trafficking; however, the specific substances transported by the vesicles require further study.

Phytohormones regulate multiple processes in plant growth and development, including mediating plant–pathogen interactions and balancing plant growth and defense. In addition to JA, salicylic acid, and ET, which are widely considered to be involved in plant immune responses, hormones such as BR, auxin, gibberellin, cytokinin, and ABA, extensively studied in plant growth and development, are also thought to directly or indirectly modulate plant responses to pathogens (Bari and Jones, 2009; Yang et al., 2015; Zhao and Li, 2021). In this study, the number and types of hormone-related DEGs in FG were higher than those in KT, including the eight previously discussed hormones. Among them, the number of DEGs belonging to the BR signaling pathway was the largest, accounting for 47.37% of hormone-related DEGs (**Figure 5**). DAMs in response to *M. rosae* were significantly enriched in the BR biosynthetic pathway in FG. Combined transcriptome and metabolome analyses revealed that DEGs in this pathway were significantly positively correlated with DAMs (**Figure 8**; **Supplementary Table 11**). After EBL treatment, inoculation with *M. rosae* enhanced the susceptibility of FG leaves (**Figure 9**), indicating that BR may negatively regulate the resistance of susceptible roses to *M. rosae*. In a study on gray mold disease in rose, BR was found to positively regulate the resistance of *Botrytis cinerea* (Liu et al., 2018). In rice, BR positively regulates disease resistance to *Magnaporthe grisea* and *Xanthomonas oryzae* pv. *oryzae* (Nakashita et al., 2003) and negatively regulates its resistance to *Pythium graminicola* and rice black-streaked dwarf virus (De Vleesschauwer et al., 2012; He et al., 2017). This indicates that even within the same species, BR regulates plant defense responses to pathogens in a complex manner and may be related to the type of pathogen and genetic background of the host. Spraying with appropriate concentrations of BR biosynthesis inhibitors, such as brassinazole, can be attempted for the follow-up prevention and treatment of rose black spot disease.

## Data availability statement

The datasets presented in this study can be found in online repositories. The names of the repository/repositories and accession number (s) can be found below: Genome Sequence Archive in the National Genomics Data Center, PRJCA015206 and PRJCA015150.

## Author contributions

JS: Formal Analysis, Funding acquisition, Methodology, Validation, Writing – original draft, Writing – review & editing. FC: Funding acquisition, Investigation, Validation, Visualization, Writing – review & editing. BL: Methodology, Resources, Writing – review & editing. CG: Formal Analysis, Investigation, Writing – review & editing. JY: Formal Analysis, Investigation, Writing – review & editing. JG: Investigation, Validation, Writing – review & editing. LH: Investigation, Validation, Writing – review & editing. GN: Conceptualization, Funding acquisition, Writing – review & editing. YY: Conceptualization, Project administration, Resources, Supervision, Writing – review & editing. FX: Conceptualization, Funding acquisition, Project administration, Resources, Supervision, Writing – review & editing.
