# Supplementary material for: Comparative transcriptome and metabolome analysis revealed diversity in the response of resistant and susceptible rose (Rosa hybrida) varieties to Marssonina rosae
Source: Front Plant Sci. 2024 Feb 22;15:1362287. doi: 10.3389/fpls.2024.1362287 (PMC10917926; doi:10.3389/fpls.2024.1362287)
Supplement: Supplementary file 1 [file DataSheet_1.pdf]

Supplementary Material

Supplementary Figures

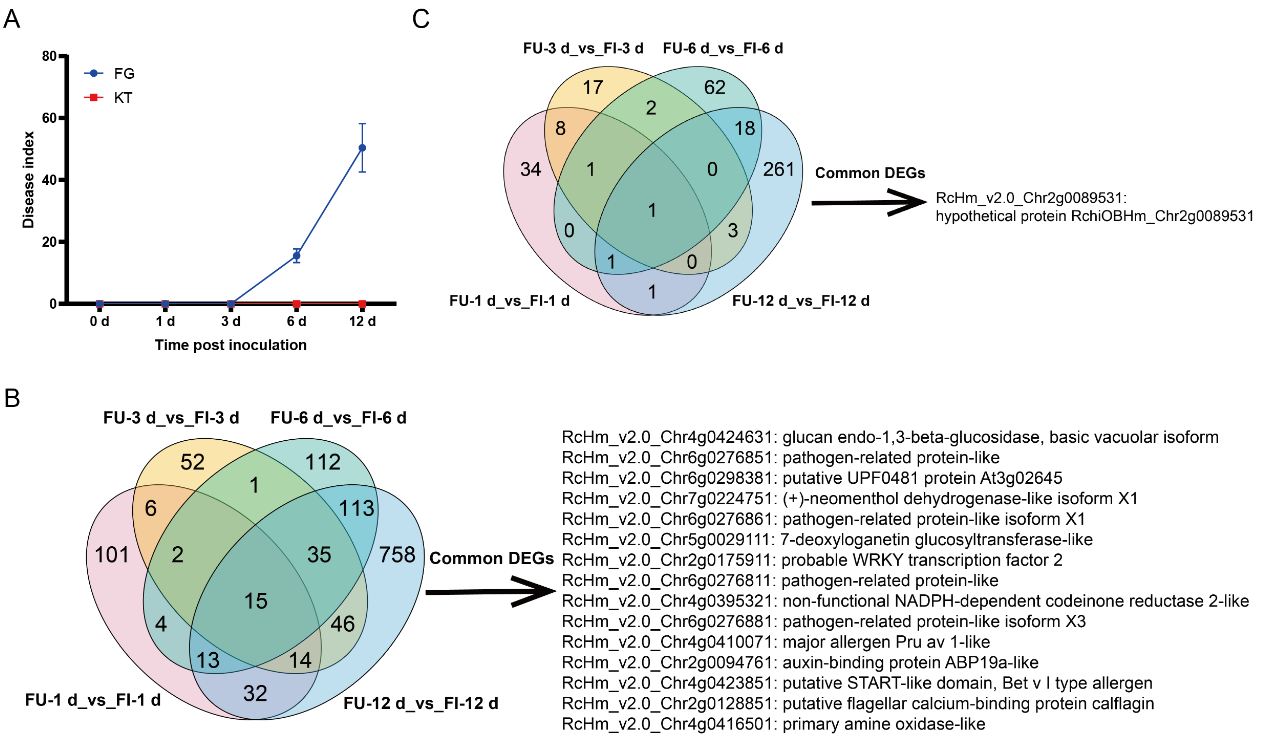

Supplementary Figure 1. Line chart of the disease index after FG and KT inoculation with *M. rosae* (A), and Venn plots of upregulated DEGs (B) and downregulated DEGs (C) in the comparison of FU vs FI. The right side of the Venn plot presents annotated information for common DEGs.

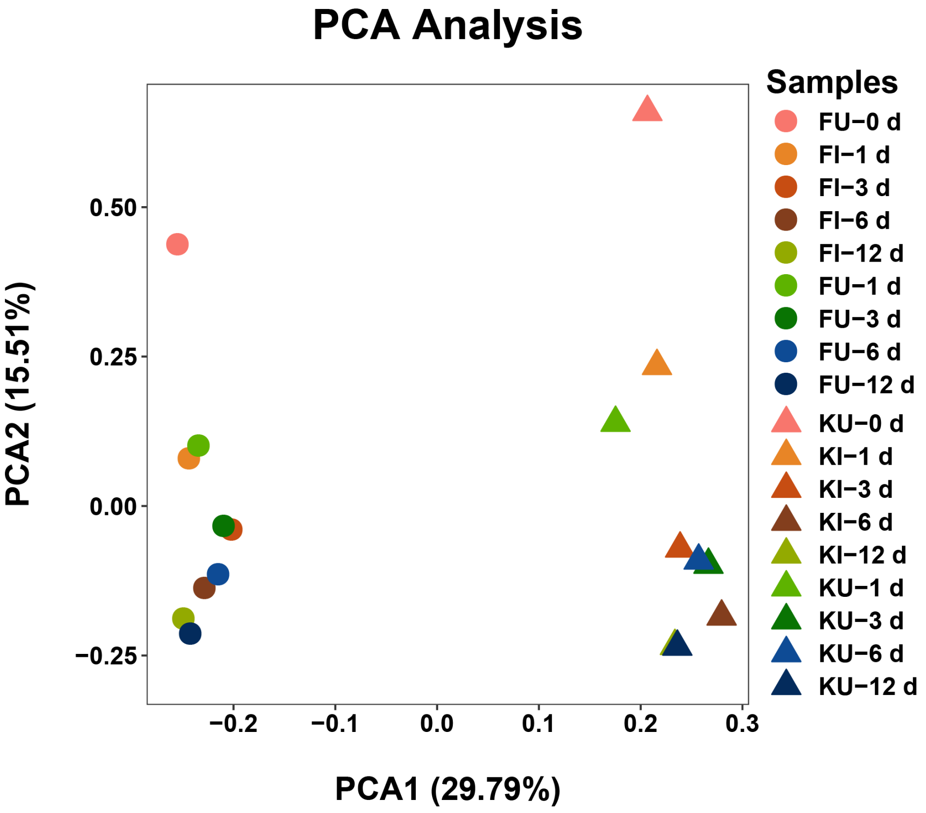

Supplementary Figure 2. Principal component analysis (PCA) of transcriptomic samples. The X and Y axes represent the first and second principal components, respectively. The percentages on the axes indicate the contribution of these principal components to the variance in the samples.

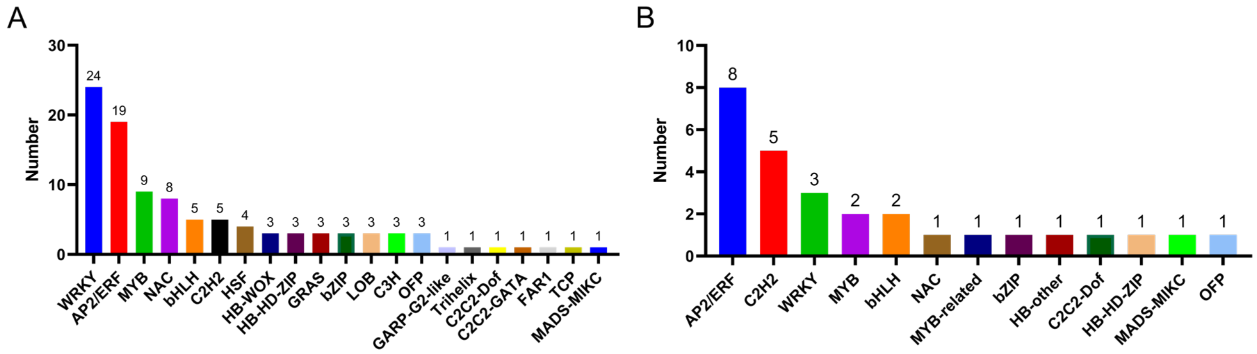

Supplementary Figure 3. Number of differentially expressed transcription factor families in FG (A) and KT (B)

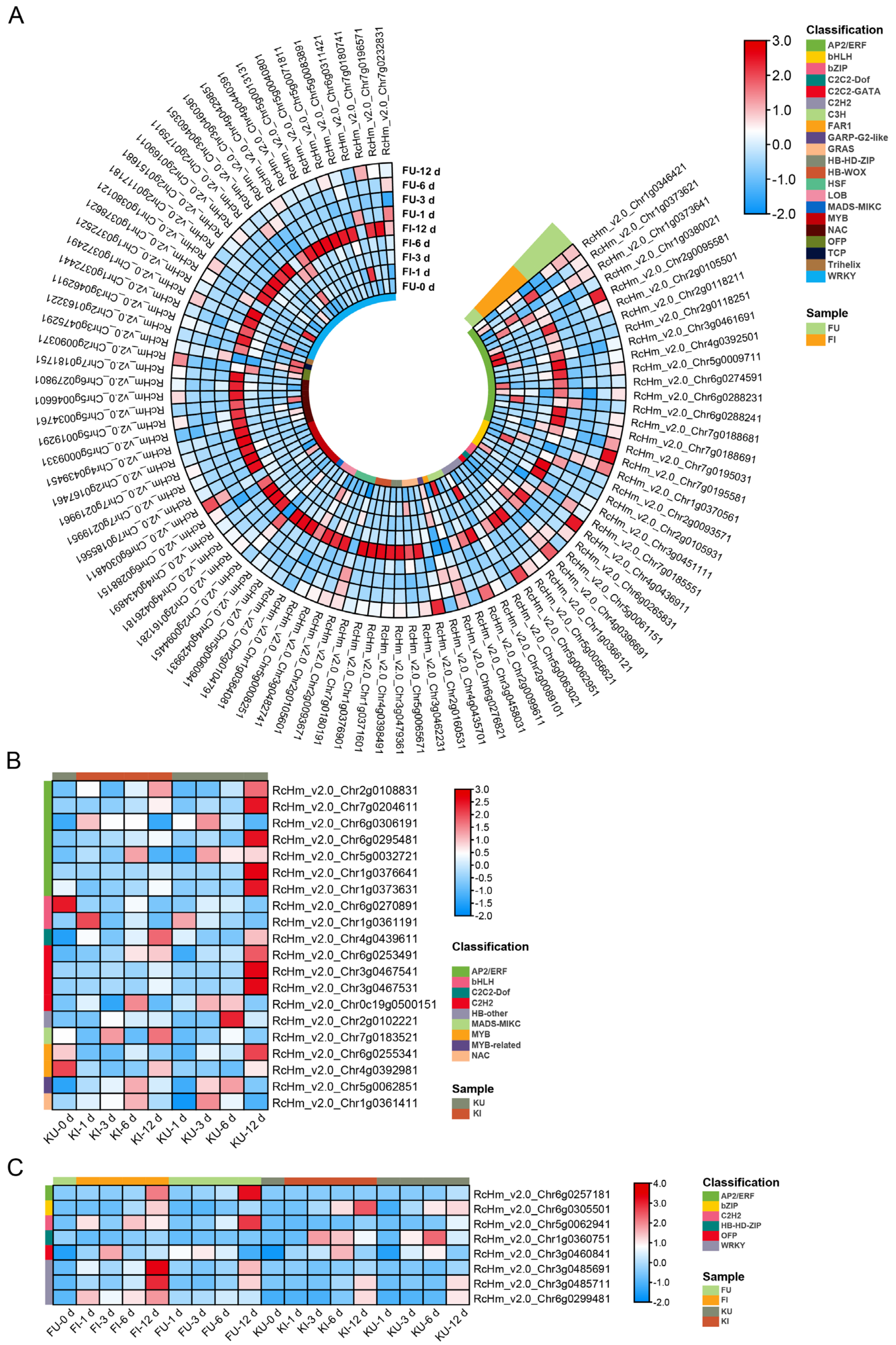

Supplementary Figure 4. Heat maps of transcription factors in response to *M. rosae* infection (RM-TFs). (A) Heat map of RM-TFs unique to FG, (B) heat map of RM-TFs unique to KT, and (C) heat map of common RM-TFs in FG and KT. The color blocks on the lateral left indicate different TFs families, and the color blocks on the transverse top indicate different types of samples.

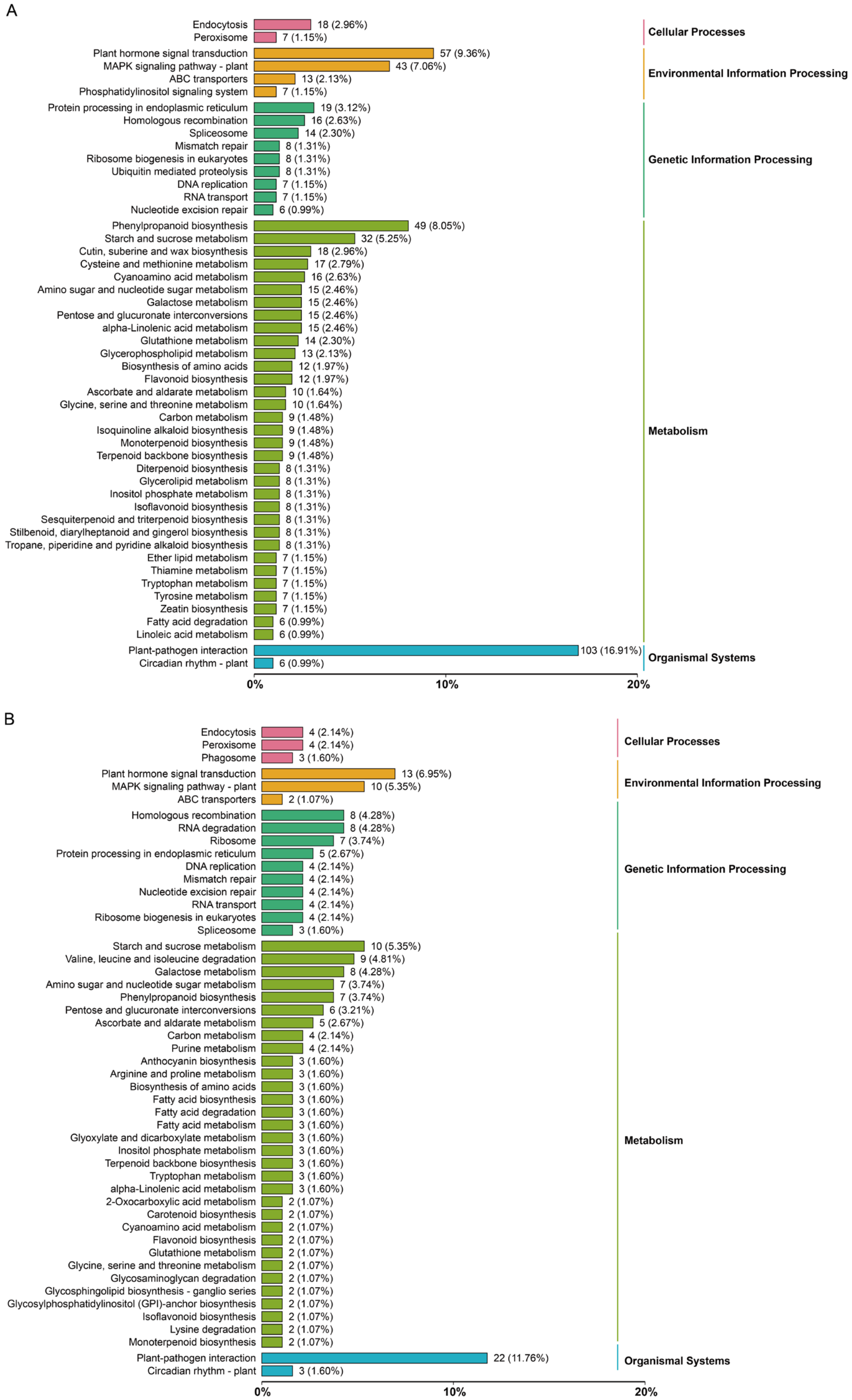

Supplementary Figure 5. KEGG classification of DEGs sets in FG (A) and KT (B). The left ordinate represents the KEGG pathway name, the right ordinate represents the first-class classification name corresponding to the annotated pathway, and the abscissa is the number of genes annotated to the pathway and its proportion to the total number of annotated genes.

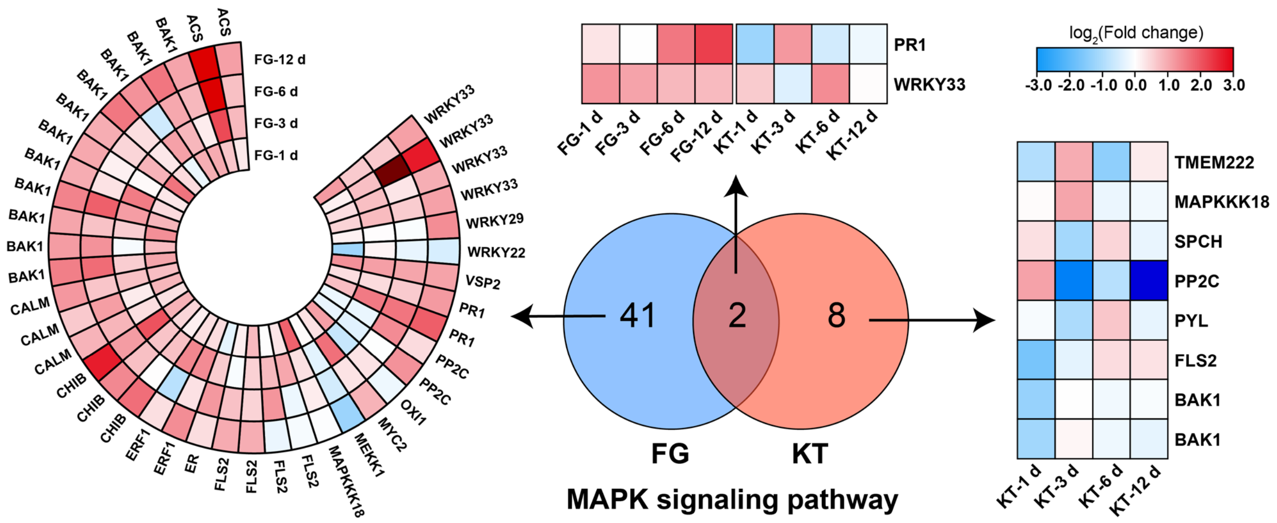

Supplementary Figure 6. Heat maps of DEGs derived from the MAPK signaling pathway. Upregulated and downregulated DEGs are indicated in red and blue, respectively.

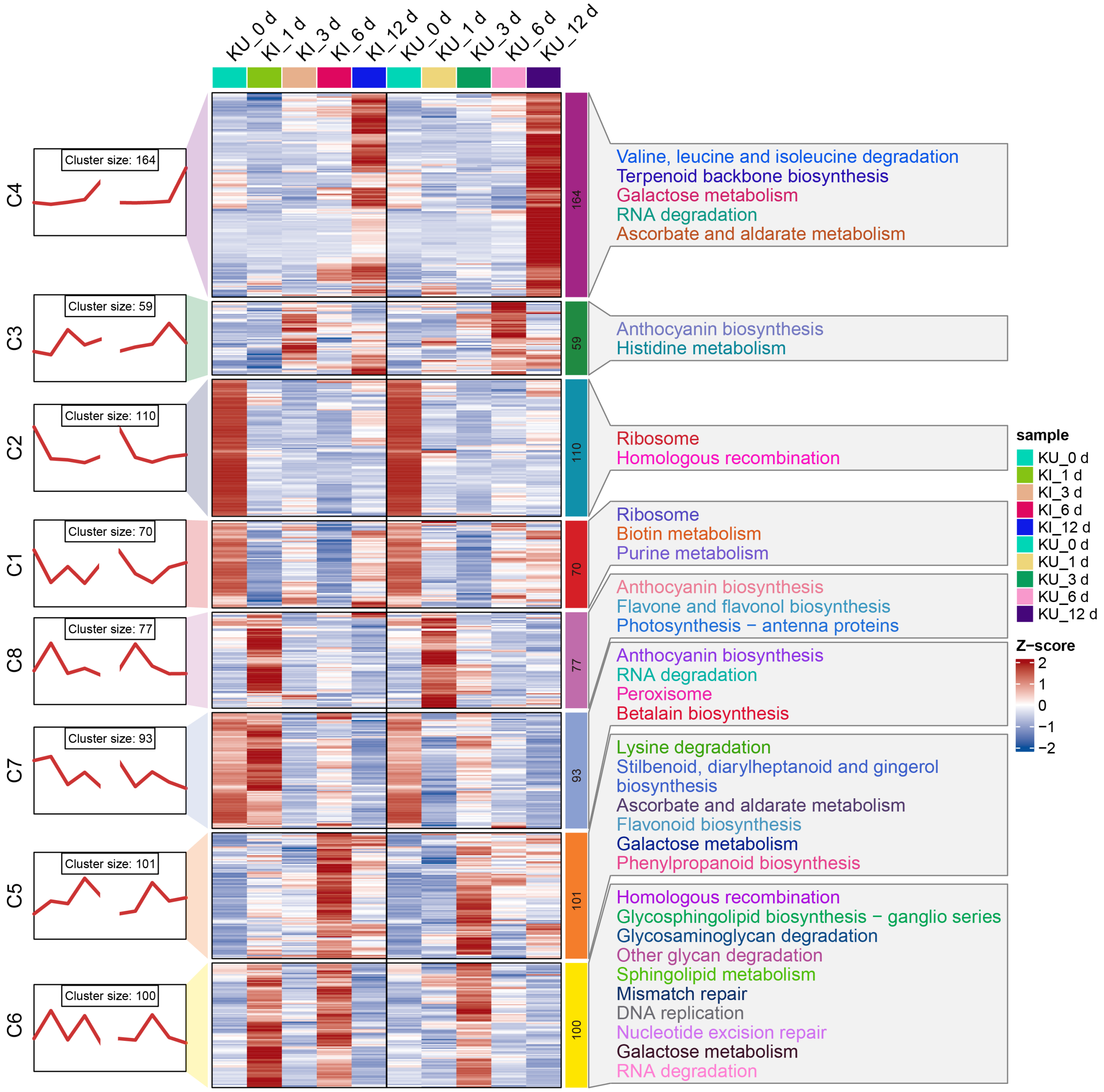

Supplementary Figure 7. Expression trends, heat maps, and significantly enriched KEGG pathways of each cluster of DEGs in KT. The left side shows a line plot of the expression trend of DEGs in each cluster of the KI and KU samples. The left side of the rectangle shows the serial number of clusters, and the number of genes within each cluster is shown above the line plot. In the middle, the heat maps of each cluster of DEGs in the KI and KU samples are shown, and changes in the color of the heat map from blue to red indicate changes in expression level from low to high. The right side shows the pathways that were significantly enriched for DEGs in each cluster after KEGG enrichment analysis.

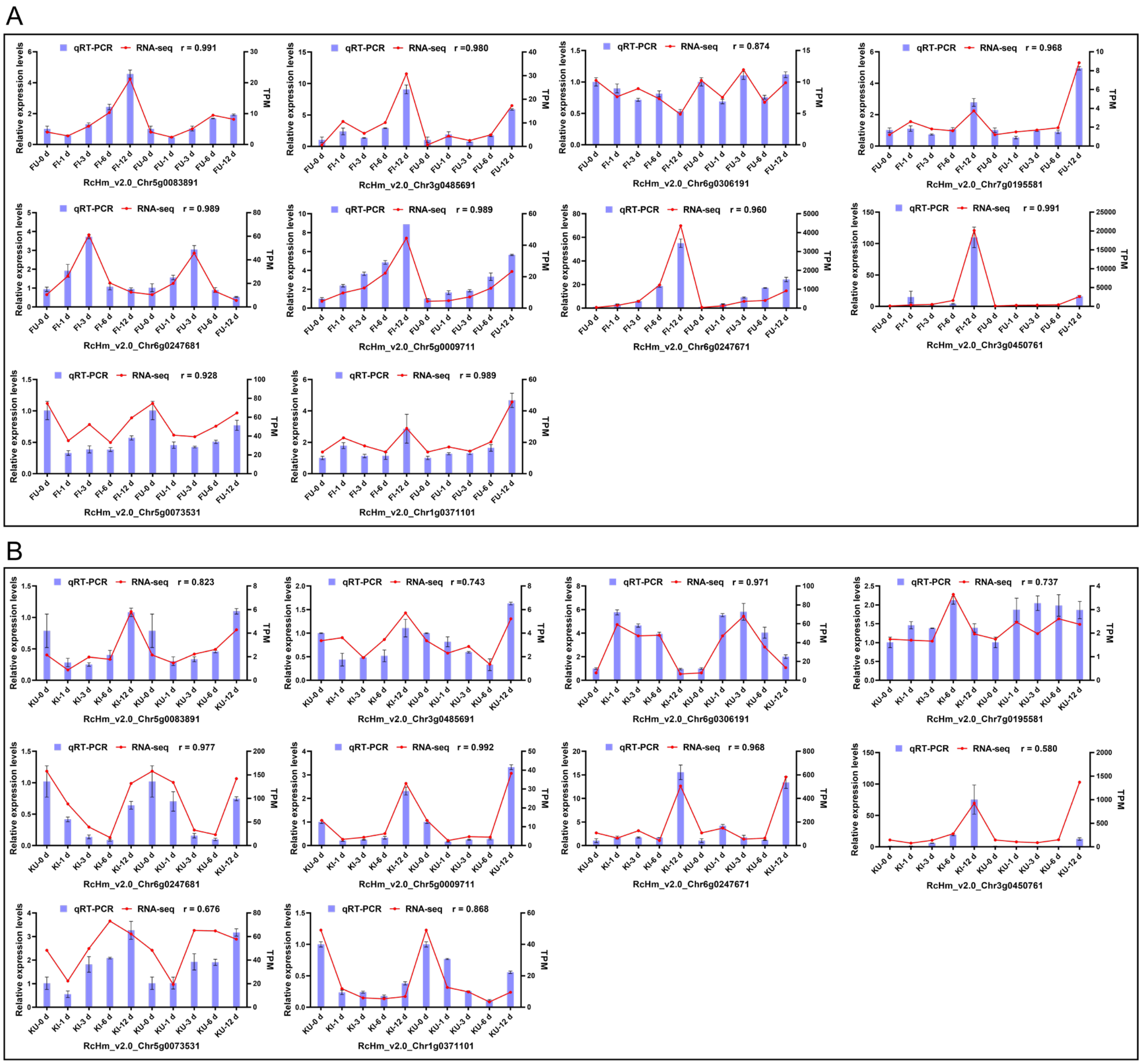

Supplementary Figure 8. Correlation between the qRT-PCR results and transcriptome data for FG (A) and KT (B). qRT-PCR results and mean TPM values of the samples at each time point are displayed in the column and line charts, respectively. Error bars represent the standard deviation, and r represents the correlation coefficient between the two sets of outcome data.

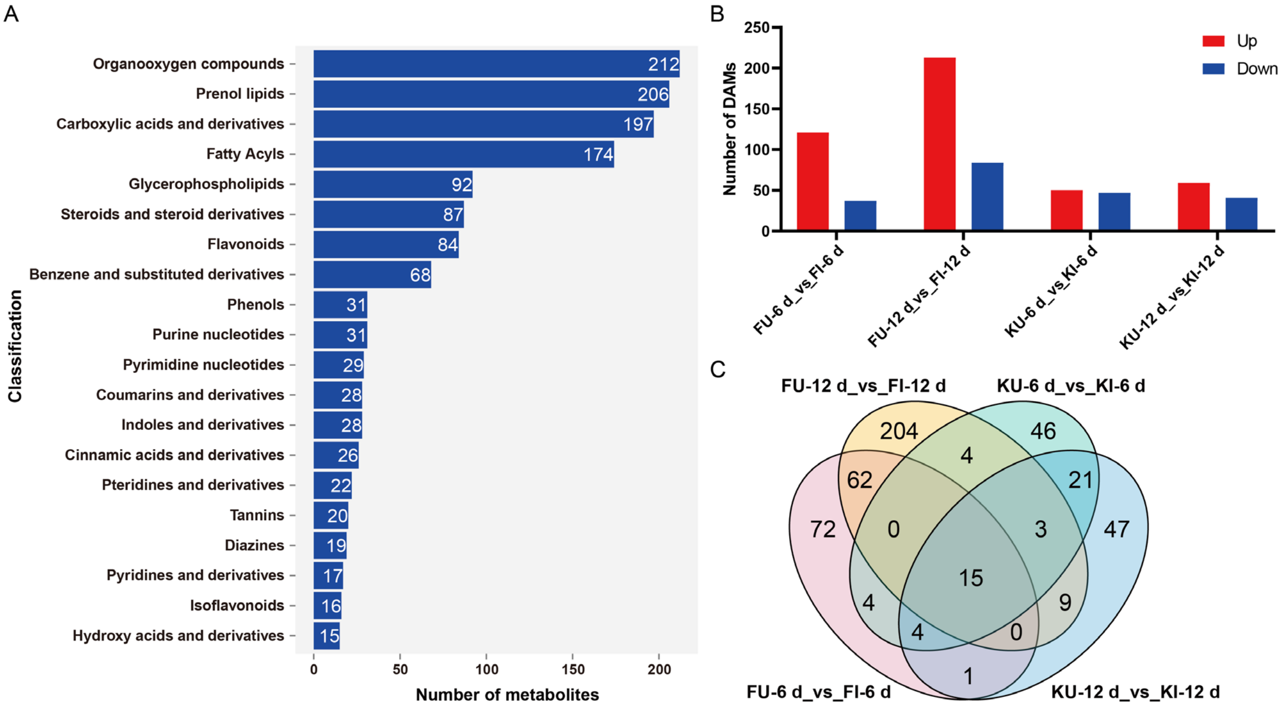

Supplementary Figure 9. Classification of metabolites detected in the two varieties (A), and number of upregulated and downregulated differentially accumulated metabolites (DAMs) in each comparison (B) and Venn plot of DAMs (C).

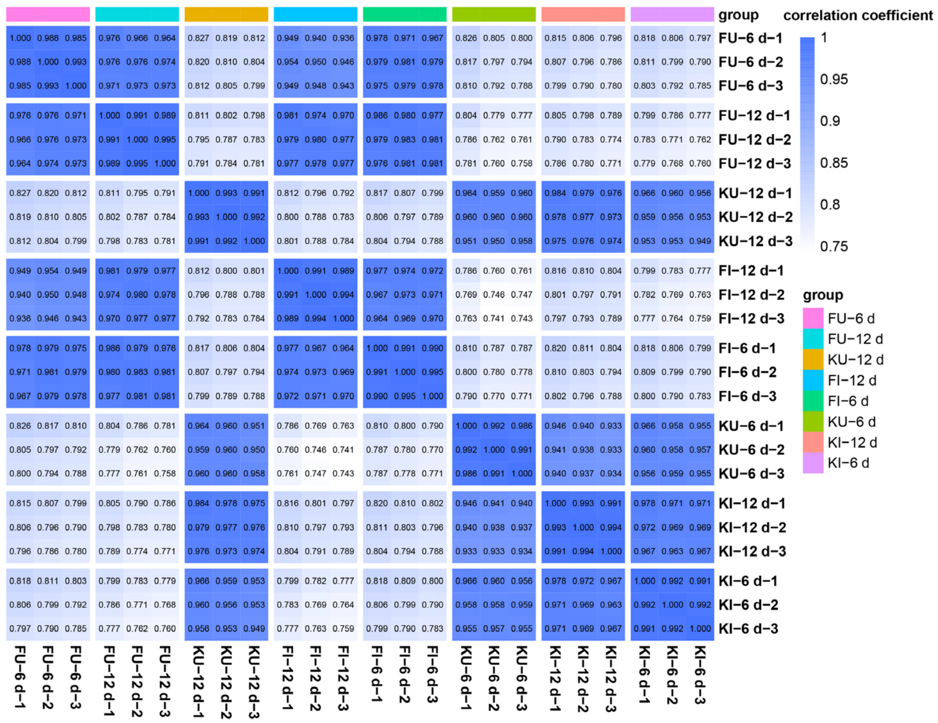

Supplementary Figure 10. Correlation heat map between metabolome samples. The deeper the blue color, the higher the correlation.

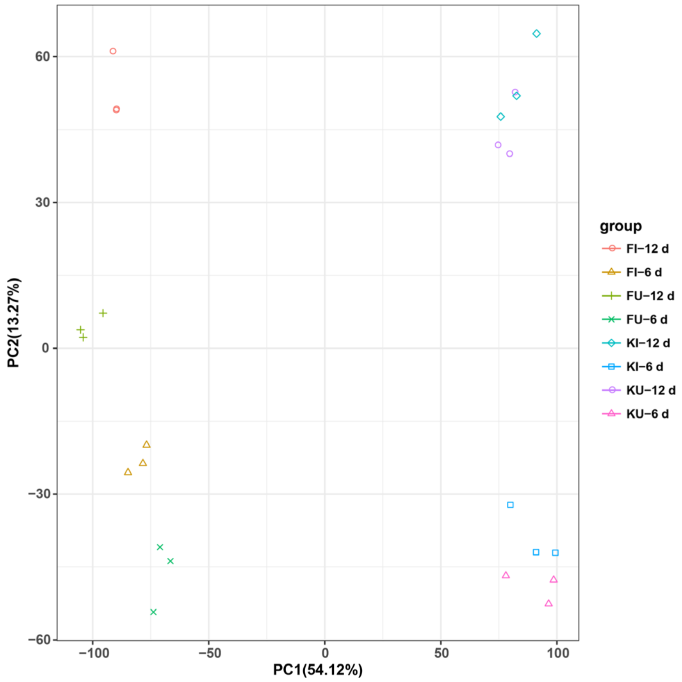

Supplementary Figure 11. PCA of metabolomic samples. The X and Y axes represent the first and second principal components, respectively. The percentages on the axes indicate the contribution of these principal components to the variance in the samples. The symbols on the right define each group, and samples with the same symbol are part of the same group.

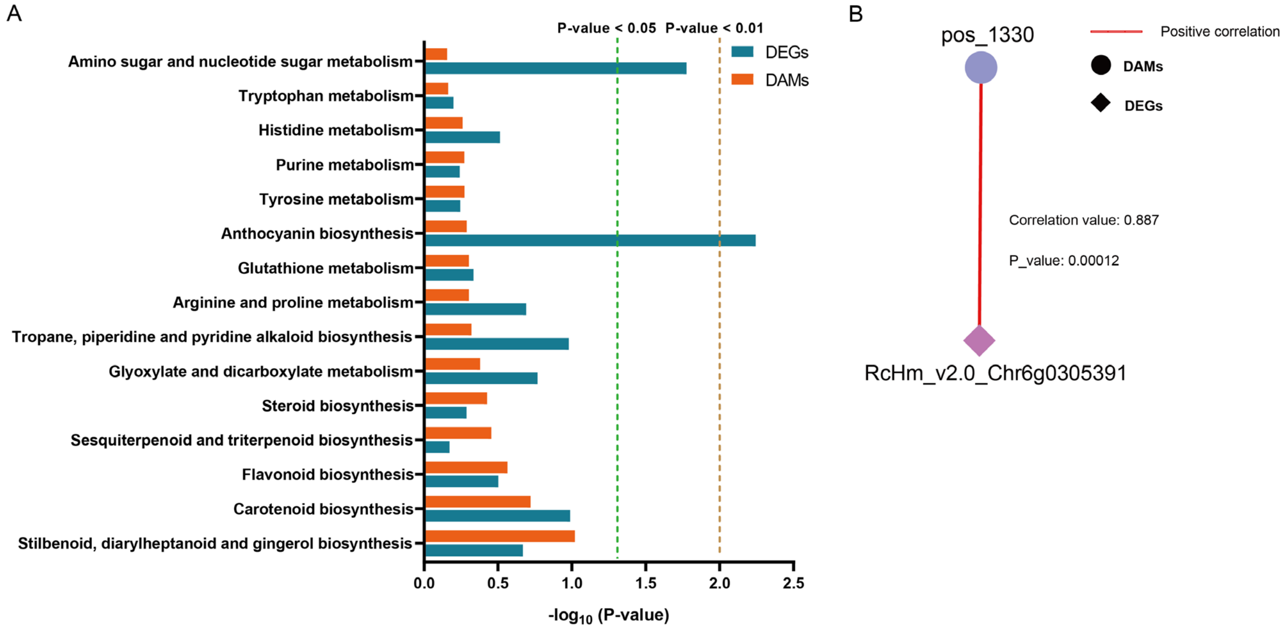

Supplementary Figure 12. Fifteen KEGG pathways co-enriched by DEGs and DAMs in KT at 6 and 12 dpi (A). Co-expression networks of DAMs and DEGs in the "sesquiterpenoid and triterpenoid

biosynthesis” pathway (B). Red line indicates a positive correlation between DAMs and DEGs, and circle and diamond indicate DAM and DEG, respectively.
